# Is There More to Olive Oil than Healthy Lipids?

**DOI:** 10.3390/nu15163625

**Published:** 2023-08-18

**Authors:** Akritas Isaakidis, Jane El Maghariki, Sérgio Carvalho-Barros, Ana Maria Gomes, Marta Correia

**Affiliations:** 1 Centro de Biotecnologia e Química Fina (CBQF)-Laboratório Associado, Escola Superior de Biotecnologia, Universidade Católica Portuguesa, Rua Diogo Botelho 1327, 4169-005 Porto, Portugaljanemaghariky@gmail.com (J.E.M.); mmcorreia@ucp.pt (M.C.); 2 Department of Nutritional Sciences and Dietetics, International Hellenic University of Thessaloniki, Sindos, 57400 Thessaloniki, Greece

**Keywords:** olive oil, human health benefits, review

## Abstract

The Mediterranean diet is a healthy dietary pattern whose main characteristic is olive oil consumption. The potential health benefits of olive oil have been extensively investigated and the present review provides the more recent clinical evidence supporting the positive impact of olive oil intake on human health. PubMed (*n* = 227) and Scopus (*n* = 308) databases were searched for published clinical studies in English over the past six years (October 2016 to December 2022), following key word searches of “olive oil” and “health”. Major findings associated olive oil with antioxidant and anti-inflammatory effects, improvement in endothelial function and lipid profile, prevention of obesity, diabetes, cardiovascular and neurodegenerative diseases, and modulation of the gut microbiota. These benefits are attributed to the nutritional composition of olive oil, which has a high content of monounsaturated fatty acids (MUFA) (oleic acid in particular) and minor compounds such as polyphenols (oleuropein and hydroxytyrosol). Although additional research continues to be required, the more recently generated evidence supports the potential of olive oil to contribute beneficially to health and to the prevention and management of a variety of non-communicable diseases, as a consequence of the synergism between its components’ complexity.

## 1. Introduction

Olive oil (OO) is a vegetable oil obtained from olives, the fruits of the olive tree (*Oleaeuropaea* L.; family Oleaceae). The cultivation of olive trees, harvest of olives, and production of OO have been linked to the history and culture of some of the most ancient Mediterranean civilizations [1,2].

Today, OO is still mainly produced and consumed in countries surrounding the Mediterranean Sea. Approximately 70% of OO production is from Mediterranean countries. The major producer worldwide is Spain, followed by Greece, Italy, Turkey, Morocco, and Tunisia [3,4]. The highest annual per capita OO-consuming countries are Greece (12 kg), Spain (11.7 kg), Italy (8.2 kg), and Portugal (7.9 kg). Northern Europe and North America consume far less, but their consumption of OO has been steadily rising [5,6].

OO is obtained by pressing and crushing whole olives and separating the oil by physical or chemical processes. If OO is obtained “solely by mechanical or other physical means under conditions, particularly thermal conditions, that do not lead to alterations in the oil, and which have not undergone any treatment other than washing, decantation, centrifugation, and filtration” it is designated as virgin olive oil (VOO) [7]. This manufacturing technique allows the preservation and transfer of bioactive components, including polyphenols, from olives to OO [8].

According to their quality, and upon international agreement in alignment with the trade standard applying to olive oils and olive-pomace oils of the International Olive Council [7], VOOs can be either classified as extra-virgin olive oil (EVOO) or as virgin olive oil (VOO). EVOO has a free acidity level, expressed as oleic acid, of not more than 0.80 g per 100 g and presents no organoleptic defects, having optimal taste and odor. It is the OO with the best quality. VOO has a free acidity level of not more than 2.0 g per 100 g and may have some minor sensory defects [7].

The composition of OO varies with the olive variety, climatic conditions, soil, irrigation, altitude, time of harvest, production techniques, and storage duration and conditions [2,9,10]. These different factors determine the OO qualitative and quantitative profiles, influencing the organoleptic/sensorial and health properties of the vegetable oil [11].

Regarding the nutritional profile, the major components of OO are fatty acids (98–99%) predominated by MUFA (55.3–86.5%; oleic acid and palmitoleic acid), followed by polyunsaturated fatty acids (PUFA) (3.5–21%; linoleic and linolenic acids), and saturated fatty acids (SFA) (8–25.1%; myristic, palmitic and stearic acids). On the other hand, minor components, which correspond to the unsaponifiable fraction, comprise between 1 to 2%, and include sterols, terpenoids and aliphatic alcohols, pigments, squalene, tocopherols (vitamin E), and polar phenolic compounds, as the most important ones [12,13]. Hydroxytyrosol and its derivatives stand out as the major phenolic compounds in VOO associated with positive health impacts. In fact, serum levels of hydroxytyrosol oleuropein and oleic acid derivatives have been proven to elevate with VOO consumption. Further research has established possible metabolic pathways affected by intake of the oil, which in turn play a role in ameliorating one’s health [13]. The historical key role of OO is well demonstrated as it has long been the basis of the Mediterranean population’s diet, becoming an essential food item of the Mediterranean diet (MedDiet) [2]. Indeed, the available evidence on OO and its beneficial health impacts has been associated with both its specific composition (fatty acids profile and phenolic compounds) as well as its consumption within the context of the Mediterranean diet. The MedDiet may slightly differ based on regional, religious, and cultural factors, but all Mediterranean countries share the use of OO as the main culinary fat. Besides OO, the MedDiet, in general, is a dietary model characterized by a high consumption of vegetables, legumes, fruits, nuts, and whole cereals; a moderate intake of fish and seafood; a low intake of meat, processed meat, and dairy products; along with a modest consumption of alcohol, mostly in the form of red wine with meals [14,15]. In the MedDiet, the OO consumption typically ranges between 25 and 50 mL (approximately two tablespoons) per day [16].

Also, when compared to other dietary treatments, the MedDiet represents a set of traditional practices and knowledge and can be considered as a way of life, rather than just food choices. For this reason, UNESCO, in 2013, inscribed the MedDiet as an Intangible Cultural Heritage of Humanity [17]. Moreover, adherence to MedDiet is associated with longevity and with a lower incidence of non-transmissible chronic diseases and health complications regarding the cardiovascular system, obesity, diabetes, and related metabolic disorders [9,18].

Important systematic review and/or meta-analysis papers have been published over the last few years concerning the benefits of the regular intake of different types of OO, or its components, on anti-inflammatory potential [19], metabolic syndrome [20], cardiovascular risk factors [21] and type 2 diabetes mellitus [22]. The clinical trials covered in these papers focused on studies performed up to 2016–2018. However, over the last years the number of trials has increased significantly, particularly since 2016, since which more than 50 clinical trials have been undertaken every year, revealing the pertinence of the topic. Based on the above, the aim of the present review is to summarize recent clinical findings performed over the past six years between October 2016 and December 2022 regarding the beneficial effects of OO consumption on human health, bringing together in the same document several ailments, and trying to pinpoint whether these are associated with the specificity of, or the synergism between, OO components.

## 2. Materials and Methods

### 2.1. Bibliographic Search

The initial literature search enabled the retrieval of 535 articles from the PubMed (*n* = 227) and Scopus (*n* = 308) databases, this number included published clinical studies in English over the past six years (October 2016 to December 2022), following key word searches of “olive oil” and “health”.

**Figure 1:**
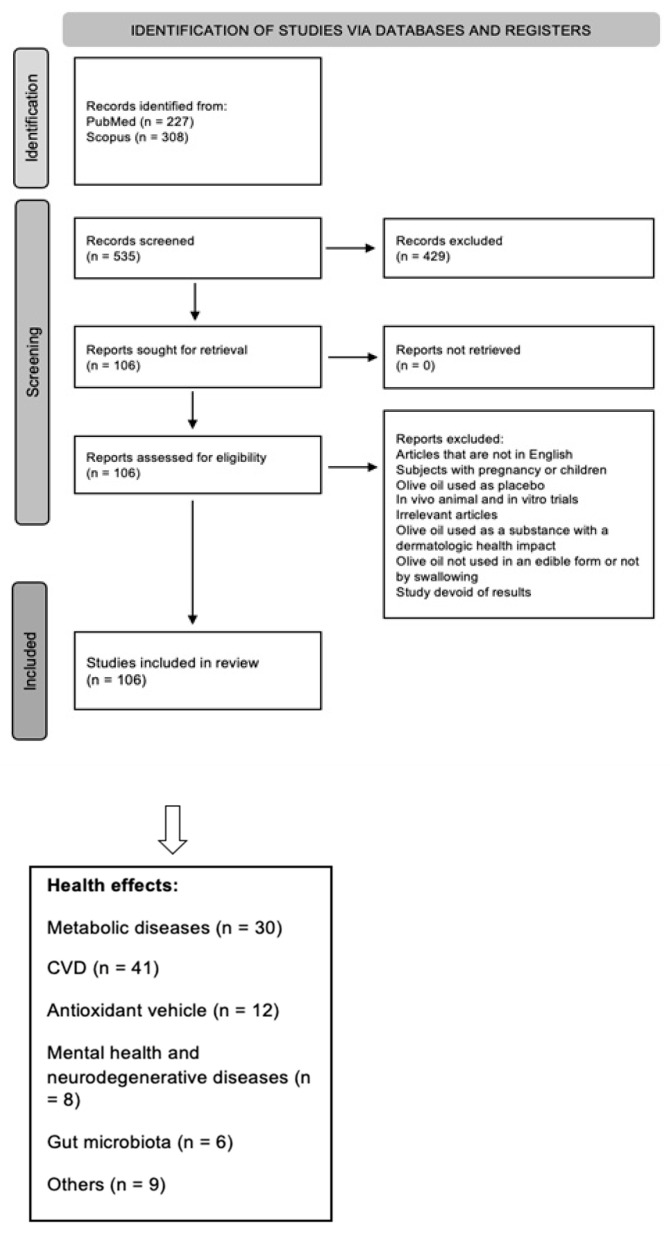
Flow chart of study selection process.

Studies should be available on the internet, in bibliographic databases of academic original articles (PubMed, Scopus). The filters applied included: Clinical Study, Clinical Trial, Clinical Trial, Phase I, Clinical Trial, Phase II, Clinical Trial, Phase III, Clinical Trial, Phase IV, and Randomized Controlled Trial. The search keywords that were selected on Scopus included: (Title) olive oil, (Title, Abstract, Keywords) clinical study. Filters applied: last 6 years, human (Figure 1).

### 2.2. Characteristics of the Excluded Studies

The established exclusion criteria included the following: (i) articles written in a language other than English; (ii) pregnant women or child participants; (iii) studies using OO as a placebo, rather than in the intervention; (iv) in vivo animal and in vitro studies; (v) duplicates; (vi) abstracts with irrelevant information or simply unsuitable intervention techniques; (vii) studies where OO was used as a substance with dermatologic health impact; (viii) studies not using OO in edible forms or administering OO by other forms rather than by swallowing; and (ix) studies that were devoid of any results. Only original articles were included in the study; hence, systematic reviews and meta-analyses were not included in the selection. Following the cross checking of the lists by three researchers, a total of 106 papers were identified. The final articles were separated into groups based on the claimed health effects. The distribution of the 106 articles among the different groups was as follows: 30/106 articles associated with “metabolism”, 41/106 articles with “CVD” (cardiovascular diseases), 12/106 articles with “antioxidants” capacity, 8/106 articles with “mental health and neurodegenerative diseases”, 6/106 articles with “gut microbiota”, and the final 9/106 articles were associated with “other health outcomes” (Figure 1).

## 3. Results and Discussion

### 3.1. Olive Oil’s Effects on Metabolic Diseases

Several factors may influence the biochemical impact of OO including components quantitative and qualitative profiles, absorption, and metabolism. In their comprehensive review, Vazquez-Aguilar et al. [13] used metabolomics to unravel available evidence from human, animal, and in vitro studies on the metabolic effects of VOO or its bioactive compounds. Despite heterogeneity between studies, the impact of regular VOO consumption on carbohydrate, lipid, and amino acid metabolism was shown, although specific metabolites could not be identified. The combined action of MUFA and polyphenol components on different metabolic pathways may contribute positively to control lipid metabolism, favor good glycemic control, and ensure good anti-inflammatory activity. Table 1 summarizes the main studies on the direct effect of OO intake on metabolic studies [23,24,25,26,27,28,29,30,31,32,33,34,35,36,37,38,39,40,41,42,43,44,45,46,47,48,49,50,51].

Few studies have shown changes in the grade of fatty liver with the consumption of OO, yet promising features have been registered. A 10-week randomized controlled trial was conducted to assess the effects of vegetable oils (canola, sunflower, and olive oils) on the lipid profile and severity of fatty liver in women with polycystic ovarian syndrome. OO consumption resulted in no significant reduction in lipid profile. However, canola and olive oils had the potential to lower considerably the fatty liver grade and Homeostatic Model Assessment of Insulin Resistance (HOMA-IR) [23]. In another study, EVOO with high oleocanthal concentration was given (32 mL/day) for 2 months to subjects with metabolic syndrome and hepatic steatosis. The ingestion of oleocanthal-enriched EVOO reduced body weight, waist circumference, body mass index (BMI), alanine transaminase, inflammatory cytokines, and hepatic steatosis [24].

Individuals who underwent a 5-year MedDiet intervention with EVOO, or nuts, exhibited slight improvements in body weight and fewer increases in waist circumference when compared to the control diet. The fact that a high-fat MedDiet has no effect on body weight and waist circumference gives support to the idea that beneficial vegetable fats should not be reduced for body weight management [25].

Even though OO has shown a positive correlation with BMI and waist circumference, AlKhattaf et al. (2020) did not identify such a relationship in a cross-sectional investigation using a Saudi adult cohort [26]. However, high OO consumers who had a significantly higher caloric intake, had a similar BMI compared to low OO consumers, indicating that OO consumption may play a role in body weight maintenance. Furthermore, a randomized controlled trial reported that results were also inconclusive in this relationship after allocating participants to one of three groups: EVOO (52 mL/day), traditional Brazilian diet (DieTBra), or DieTBra+EVOO (52 mL/day) for 12 weeks. The DieTBra is a healthy dietary pattern characterized by the consumption of rice, beans, a small amount of low-fat meat (red meat, chicken, or fish), and raw and cooked vegetables in the main meals (lunch and dinner); consumption of fruits, bread, milk, and dairy products in the small meals; and low consumption of processed foods. The authors observed that people in the DieTBra group had large decreases in total body fat and body weight, as well as significant gains in walking speed and handgrip strength (sarcopenia indicators) in severely obese adults. There was also a substantial reduction in body weight in the DieTBra+EVOO group when compared to the EVOO-only group; however, EVOO alone did not enhance any of the above cited outcomes [27].

Additionally, OO seems to mobilize some hormones and indicators important in mitigating obesity. Monfort-Pires et al. (2021) conducted a short-term dietary intervention and found that EVOO ingestion was able to induce significant increase in blood levels of MUFA, leptin, secretin, fibroblast growth factor 21 (FGF21), and 12,13-dihydroxy-9Z-octadecenoic acid (12,13 di-HOME) in lean volunteers [28]. Increase in leptin concentration was associated with increased brown adipose tissue activity. Also, secretin, FGF21, and 12,13di-HOME are inducers of brown adipose tissue activity, providing the first evidence that EVOO leads to an increase of brown adipose tissue activity in humans.

Recall that omentin and adiponectin have a protective role in preventing obesity-related comorbidities. In this context, Kabiri et al. [29] conducted a randomized crossover trial with overweight women and found that an OO-rich diet tended to increase omentin and adiponectin levels in comparison with a hypocaloric diet, showing a putative role for OO in preventing obesity.

In another study, Martinussen et al. (2020) discovered that combining long-chain fatty acids with 2-oleoyl-glycerol (2-OG) (released during digestion of 20 mL of OO) was significantly more effective in stimulating enteroendocrine secretion and that distal, but not proximal, gut hormones were increased in Roux-en-Y gastric bypass (RYGB) patients [30]. Gut hormones induced by fat consumption are essential regulators of energy and glucose homeostasis and may play a role in weight reduction and diabetes remission after RYGB.

In a tentative approach to determine the acute effects of the addition, or substitution, of fat and carbohydrates with whey protein-rich liquid supplements, which may be used in weight control, on gastric emptying and other neuroendocrine parameters, Giezenaar et al. (2018) conducted a randomized, double-blind trial, including 13 healthy young men who ingested a control drink or iso-volumetric drinks containing different ratios of protein/carbohydrate/fat [31]. The equi-energetic substitution of whey protein with dextrose and OO (12.4 g) resulted in faster gastric emptying, less ghrelin suppression, and less stimulation of cholecystokinin (CCK) and glucagon-like peptide-1 (GLP-1) concentrations; however, adding the dextrose and OO to whey protein did not further slow gastric emptying, suppress ghrelin, or increase CCK and GLP-1 responses of the drink [31].

When it comes to nonalcoholic fatty liver disease (NAFLD) few studies could be found on potential benefits of OO. A subgroup analysis within the PREDIMED (PREvención con DIeta MEDiterránea) trial, a long-term prospective, parallel, randomized, controlled, multicenter trial designed to assess the effects of the MedDiet on the primary prevention of CVD, found that long-term adherence to a MedDiet + EVOO is associated with a lower prevalence of NAFLD in older people at high cardiovascular risk, when compared to a similar diet supplemented with nuts or a low-fat control diet [32]. In another independent study, patients with NAFLD were given 20 mL of OO or sunflower oil (SFO) daily for 12 weeks. The OO consumption group were able to register increased skeletal muscle mass while lowering serum triacylglycerols, fat mass, body fat percentage, and fatty liver grade. Therefore, in people with NAFLD, OO may reduce the severity of the condition without modifying cardiometabolic risk factors [33]. In a trial of individuals with NAFLD, a reduced calorie diet fortified with VOO (20% of the total fat (30%)), along with minor weight loss, reinforced the anticipated effects of weight loss in reducing the levels of the hepatic alanine aminotransferase (ALT) and aspartate aminotransferase (AST) enzymes. In addition, a drop in intrahepatic lipid content was seen between the baseline and after the VOO dietary intervention [34].

Several trials have been conducted concerning the effect of OO consumption on postprandial blood glucose (PBG) modulation. Carnevale et al. (2017) examined whether EVOO affects PBG and lipid profiles in patients with impaired fasting glucose (IFG) and discovered that a meal containing 10 mL of EVOO was associated with a reduction in glucose and dipeptidyl-peptidase-4 (DPP4) activity, a significant decrease in triacylglycerols (TG) and apolipoprotein B-48 (Apo B-48), and a significant increase in insulin and GLP-1 [35]. A study was performed on patients with type 1 diabetes (T1D) consuming three high-glycemic index meals differing in fat types one week apart; it was shown that blood glucose was lower after the EVOO than after the butter or low-fat meals. EVOO also significantly increased gastric emptying, GLP-1 secretion, and triglyceride concentration. These results suggest a hypoglycemic effect of EVOO in T1D patients, which may be useful in their treatment [36].

Different results were demonstrated in randomized controlled crossover research, aiming at studying the effect of EVOO on PBG in Asian type-2 diabetes (T2D) patients. They found a significant rise in two-hour PBG in patients consuming EVOO versus patients having meals without EVOO. Because free fatty acids impeded glucose transport and insulin secretion, adding EVOO to meals gave no significant benefit on PBG [37].

In a randomized control trial (RCT), 34 adults, aged 18–64 years with T2D and class II/III obesity, completed the intervention after being randomized into two groups: EVOO and EVOO+DieTBra. Significantly reduced fasting insulin levels were observed in the group who were also consuming the DieTBra, noting that the OO intake was almost the same between the groups. The EVOO+DieTBra groups also showed decreased BMI, weight, serum levels of inflammatory cytokines, interleukin-1α (IL-1α), and adiponectin, as well as increased tumor necrosis factor α (TNF-α). This study concluded that EVOO+DieTBra has a role in ameliorating the inflammatory profiles and fasting insulin levels of adults having class II/III obesity and T2D [38].

Interestingly, the potential efficacy of OO on glycemic management and lipid profiles was compared to that of other vegetable oils, namely rice bran oil (RBO) [39] and palm olein [40]. Independently of the study, no significantly different effects between OO and the other vegetable oils were found. In the study with RBO, 10 individuals with T2D were given 15 mL of EVOO or RBO daily for four weeks, yet changes in fasting blood glucose (FBG), PBG, total cholesterol (TC), low-density lipoprotein cholesterol (LDL-C), and TG levels were not substantially different between the two groups. In contrast, high-density lipoprotein cholesterol (HDL-C) levels declined dramatically in both groups. [39]. In the case of palm olein, a dietary crossover trial was conducted where 120 participants were recruited and randomly divided into two groups (palm olein or OO). Each participant was provided with 48 g of test oil per day. Palm olein and OO had no recognizably different effects on body fatness or blood lipids in a healthy Chinese population [40].

Mandøe et al. (2018) designed a C4-dietary oil (1,3-di-butyryl-2-oleoyl glycerol) as a 2-OG-generating fat type, which would stimulate incretin release to the same extent while providing less calories than OO [41]. Overweight patients with T2D ingested C4-dietary oil and that resulted in enhanced secretion of GLP-1 and glucose-dependent insulinotropic polypeptide (GIP) to almost the same extent as OO, despite the release of both 2-OG and oleic acid, which also may stimulate incretin secretion from OO. Thus, from this study C4-dietary oil seems to be more effective as an incretin releaser than OO itself [41].

Focusing more specifically on the OO minor bioactive compounds themselves, to eventually pinpoint structure–function relationships, interesting results were found in a crossover design in which T2D patients and healthy subjects were given either 40 g oleuropein (4 mg%)-enriched chocolate by EVOO addition or 40 g control chocolate spread. The study showed that using EVOO as a source of oleuropein administration is associated with a modest increase or no change of glycemia in T2D and healthy subjects respectively, via an incretin-mediated mechanism [42]. In another study (a double-blind, crossover, randomized controlled trial) where the same EVOO delivery vehicle was used with young healthy subjects, results showed that a short-term (2 weeks) consumption of an EVOO-enriched chocolate spread decreased circulating harmful sphingolipids and limited detrimental effects on insulin sensitivity compared to a palm oil-enriched chocolate spread [43].

One of the most important targets in nutrition is to be able to prevent the onset of non-communicable chronic diseases such as diabetes. Although previously discussed clinical trials revealed a potential impact form regular OO intake on glycemic management in T2D patients, the most desired effect would undoubtedly be prevention capacity. In this perspective, several recent research studies were found to show significant effects of fortified OO consumption on prediabetics. The results of the PREDIABOLE (PREvention of DIABetes with OLEanolic acid) trial demonstrated that regular consumption of an oleanolic acid (OA)-enriched OO substantially reduced the risk of developing T2D in prediabetic patients [44]. A short-term (12 weeks) diet rich in MUFA from OO decreased hepatic fat and improved both hepatic and total insulin sensitivity in people with prediabetes [45].

Within the PREDIMED context, promising evidence was also found among trial participants in this dominion. In one study, Basterra-Gortari et al. (2019) showed that a MedDiet supplemented with EVOO (1 L/wk) may delay the introduction of glucose-lowering medications (oral or injectable) in T2D individuals [46]. In another study, elevated baseline levels and increases in branched-chain amino acids (BCAAs) and aromatic amino acids (AAAs) were associated with an increased risk of T2D. After one year of intervention, a MedDiet high in EVOO (4 tablespoons/day) considerably lowered BCAA levels and mitigated their negative effect [47].

D’Amore et al. (2016) demonstrated that, in healthy subjects, acute consumption of polyphenol-rich EVOO was able to improve glycemia and insulin sensitivity, as well as modulate the transcription of genes and miRNAs involved in metabolism, inflammation, and cancer, modifying peripheral blood mononuclear cells to a less deleterious inflammatory phenotype [48]. In the PREDIMED trial, the results demonstrated that MedDiet treatments enriched with nuts and EVOO regulate exosomal RNA content [51].

A trial done on healthy British men and women comparing EVOO, coconut oil, and butter found that butter significantly increased LDL-C, TC/HDL-C ratio, and non-HDL-C and that coconut oil significantly increased HDL-C, but there were no significant differences on weight, fat distribution, and metabolic markers among any of the three dietary fats [49]. Another study, with a young and healthy Chinese population, compared EVOO with palm olein and cocoa butter; unexpectedly, subjects who consumed palm olein had significantly lower serum TG concentrations than those who consumed EVOO. All the other lipid indices, LDL subfractions, physical indicators, glycometabolic indices, and inflammatory indicators showed no significant differences amongst the three fats [50].

### 3.2. Olive Oil Effects on Cardiovascular Diseases (CVD)

The potential cardioprotective benefits of OO consumption have been demonstrated in several studies and are summarized in Table 2 [52,53,54,55,56,57,58,59,60,61,62,63,64,65,66,67,68,69,70,71,72,73,74,75,76,77,78,79,80,81,82,83,84,85,86,87,88,89].

The ATTICA Study was a prospective, population-based study conducted in Athens involving 3042 CVD-free adults (1514 men and 1528 women) and various dietary habits, consumption of olive oil and other fats/oils were recorded. In 2011–2012, the 10-year study follow up was performed, recording the fatal/non-fatal CVD incidence in 2020 participants (mean follow-up duration: 8.41 years). Interestingly, the ATTICA study found an inverse association between exclusive OO intake and the risk of developing CVD, with fibrinogen plasma levels showing a the most prominent mediation effect on this association [52]. Another important large cohort, analyzed two large prospective cohorts of US men and women (*n* = 92,978), followed up for 24 years, and has shown that replacing margarine, butter, mayonnaise, and dairy fat with higher OO consumption (defined as >7 mL/day or >0.5 tablespoon/day) entailed a significantly lower risk of coronary heart disease and CVD [53].

Additionally, other studies suggest that OO may have anti-inflammatory properties. In patients with cardiovascular risk factors (such as hypertension, dyslipidemia, or diabetes) referred for coronary angiography, the consumption of OO was more effective in reducing the level of inflammatory cytokine interleukin-6 (IL-6), compared to canola oil (CO) [54]. Also, in a subset of participants, higher OO intake was associated with lower levels of circulating inflammatory biomarkers and a better lipid profile [53].

OO can take part in the prevention and treatment of dyslipidemia in individuals with T2D since a combination of OO with garlic powder significantly normalized the serum cholesterol and serum TG levels in these patients [55]. Another study, performed with elderly subjects, showed that EVOO enhanced the cholesterol efflux capacity (CEC) of HDL-C to normal levels and prevented the age-related shifts of the distribution of HDL-C subclasses, through an increase in large HDL-C and a decrease in small HDL-C particles [56]. However, in patients with stable coronary artery disease, there were no significant differences in LDL-C levels [57] nor in plasma fatty acids [58] following the consumption of a healthy diet supplemented with EVOO or pecans.

Three studies showed that OO may be beneficial in reducing elevated blood pressure [59,60,61]. The regular consumption of an EVOO rich in phenolic compounds (366 mg/kg PC) by healthy individuals was found to reduce the systolic blood pressure and maintain the diastolic blood pressure compared to pre-intervention values as well as an EVOO low in phenolic compounds (2.7 mg/kg PC), by down regulation of the angiotensin I-converting enzyme (ACE), nuclear receptor subfamily 1, group H, member 2 (NR1H2) and Interleukin 8 receptor alpha (IL8RA) gene expressions, blood pressure-related genes involved in the renin–angiotensin–aldosterone system [59]. In a randomized controlled trial, daily consumption of the same amount of EVOO (25 mL/day) associated with an energy-restricted Western diet, for nine weeks, decreased the diastolic blood pressure and reduced body fat in overweight women [60]. Moreover, OO supplementation (3 g/day) reduced the resting systolic and diastolic blood pressure in young healthy men and women [61]. It also reduced muscle sympathetic nerve activity, which is linked to left ventricular hypertrophy, insulin resistance, and arrhythmogenesis when elevated [61].

Regarding blood pressure control, a MedDiet supplemented with EVOO for one year was shown to reduce both systolic and diastolic blood pressure values in women with moderate hypertension [62].

OO, rich in polyphenols, can also improve endothelial function. Three VOOs, differing in their bioactive compound (PC and triterpenes) contents (PC varied between 124 and 490 mg/kg and triterpenes varied between 86 and 389 mg/kg), were given to healthy adults; plasma endothelin-1 levels, an endothelial function biomarker linked to the development of hypertension, were reduced [63]. One study with adults at risk of T2D (either prediabetes or with metabolic syndrome) showed that the acute ingestion of high-polyphenolic EVOO (189 ppm biophenols) improved endothelial function, and therefore reduced the cardiovascular risk of the subjects, unlike refined OO (ROO) without polyphenols [64]. These findings were confirmed by two other studies reporting that EVOO, added to chocolate [65] or to a single high glycemic index meal [66], significantly increased the endothelial function and lowered the risk of CVD in T2D [65] and T1D patients [66].

The consumption of OO may have antithrombotic properties. Both EVOO and ROO intake improved several cardiovascular risk markers [lower red blood cell count, decreased erythrocyte sedimentation rate (ESR), and increased mean platelet volume] in patients with fibromyalgia [67]. Furthermore, it has been found that oral supplementation for one year with EVOO enriched with vitamins (K1, D3, and B6) could be a useful tool to prevent ischemic stroke because it reduced platelet aggregation among post-menopausal women [68]. Also, a more frequent OO intake was associated with lower platelet activation in obesity thus reducing CVD risk [69].

A recent study investigated the effect of EVOO with different polyphenol content on the postprandial modulation of cardiovascular-related microRNAs (miR) related to CVD. Twelve healthy participants consumed 30 mL of EVOO containing low (L-EVOO; 250 mg total phenols kg^−1^ of oil), medium (M-EVOO; 500 mg total phenols kg^−1^ of oil), and high (H-EVOO; 750 mg total phenols kg^−1^ of oil) enriched EVOOs. During the postprandial state, the levels of let-7e-5p decreased with EVOO regardless of polyphenol content, suggesting a general response to the fatty acid composition of EVOO; the miR-17-92 cluster increased by low and medium polyphenol content, suggesting a role in fatty acid metabolism and nutrient sensing. Thus, postprandial modulation of circulating microRNAs levels could be a potential mechanism for the cardiovascular benefits associated with EVOO intake [70]. In another study it was proposed that an acute dietary supplementation with EVOO increased runners’ cardiorespiratory coordination during a progressive walking test at moderate intensity, although it did not change performance or other physiological markers [71].

In the PREDIMED study, where the effects of the MedDiet on the primary prevention of CVD, were assessed, a total of 7447 Spanish men (aged 55–80 years old) and women (aged 60–80 years old) without diagnosis of CVD but at high cardiovascular risk were enrolled, assigned to one of the three nutritional intervention groups—MedDiet supplemented with EVOO, MedDiet supplemented with nuts, or a low-fat control diet—and were followed up for a median of 4.8 years. The main findings of the trial showed that, in comparison to the low-fat control diet, the MedDiet supplemented with either EVOO or with nuts significantly reduced the incidence of major cardiovascular events, including acute myocardial infarction, stroke, and death for cardiovascular cause [72]. On the other hand, no effect on heart failure risk (lowering capacity) was noted for these diets. However, this study may have been underpowered to provide valid conclusions and further trials are needed to better assess the effect of the MedDiet on heart failure risk [73].

In another study, nested in PREDIMED, the association between plasma ceramides, the risk of CVD, and the MedDiet was investigated. The results showed a strong positive association between plasma ceramide concentrations and CVD risk. It was also shown that the MedDiet may mitigate the potential deleterious effects of elevated plasma ceramide concentrations on CVD. These findings suggest that plasma ceramides may serve as markers of future CVD risk in clinical practice and strengthen the evidence of the cardioprotective effect of the MedDiet [74].

Hernáez et al., in two different substudies from the PREDIMED trial, showed that one year of adherence to a MedDiet enriched with VOO converts LDL particles into less atherogenic ones and improves several HDL functions (CEC, cholesterol metabolism, antioxidant/anti-inflammatory properties, and vasodilatory capacity) in individuals at high cardiovascular risk. The development of less atherogenic LDL and improved HDL functions could contribute to explaining some of the cardioprotective benefits of the MedDiet [75,76]. Indeed, the authors have shown that adherence to a traditional MedDiet, particularly when enriched with VOO, decreased LDL atherogenicity in high CVD risk individuals [90]. Another study, also by Hernáez et al. [90], reported that increases in the consumption of VOO, nuts, legumes, whole grains, and fish, achievable through a regular diet, may lead to relevant improvements in HDL functions in high cardiovascular risk subjects. These results confirm the beneficial effects of VOO, nuts, and fish in HDL function and describe for the first time an association between incrementing the consumption of legumes and whole grains and improvements in the HDL function, reinforcing the idea that a healthy diet may promote HDL functionality.

A case-cohort design, within PREDIMED, revealed that MedDiet interventions, supplemented with EVOO or nuts, for one year induced some significant changes in the lipidome although they were not significantly associated with subsequent CVD risk. It was also observed that, at baseline, lipid metabolites with a longer acyl chain and a higher number of double bonds were significantly and inversely associated with the risk of CVD [77,78].

A recent cross-sectional analysis aimed to ascertain the relationship between OO intake and ankle-brachial index in a Mediterranean population at high cardiovascular risk. The ankle-brachial index is recommended as a non-invasive tool for the screening and diagnosis of peripheral artery disease. The results suggested that the consumption of VOO promoted a better ankle-brachial index, instead of other OO categories or olive-pomace oil, which may be beneficial for peripheral artery disease prevention [78].

In a case-control study nested in the PREDIMED study, 167 peripheral artery disease cases were matched with 250 controls. Participants in the MedDiet+EVOO group were protected against peripheral artery disease regardless of baseline threonine which can be an early biomarker of future disease incidences in high-risk CVD individuals [79].

The VOO and HDL Functionality (VOHF) study is a randomized, controlled, double-blind, crossover clinical trial with 33 hypercholesterolemic volunteers, aged 35–80 years. For 3 weeks, preceded by a 2-week washout period, participants ingested 25 mL/day of three VOOs differing in the PC concentration and origin: a VOO naturally containing 80 ppm PC (VOO), a phenol-enriched VOO containing 500 ppm PC from OO (FVOO), and a phenol-enriched VOO containing 500 ppm PC from OO and thyme (FVOOT). Post hoc analyses from the VOHF study found that VOO ingestion increased HDL monolayer fluidity, increased apolipoprotein A-I concentration, and decreased HDL oxidative status, which are main determinants with CEC enhancement, which, in turn, is inversely associated with cardiovascular risk. This work points out new targets to ameliorate HDL function through nutritional interventions [80].

In a subsample of 12 hypercholesterolemic adults, from the VOHF study, the ingestion of FVOOT decreased blood oxidized-LDL (ox-LDL) concentrations [81]. In another VOHF study, both phenol-enriched OO (FVOO and FVOOT) increased HDL antioxidant content, but α-tocopherol, the main HDL antioxidant, only increased after the FVOOT intervention [82]. These results indicate that OO enriched with PC could be a useful dietary tool to obtain an additional protective effect against the atherosclerotic process in high cardiovascular risk individuals.

All these results highlight the clinical importance of OO consumption in CVD prevention and provide further evidence for health policy makers to design appropriate dietary strategies for the general population.

Jimenez-Torres et al. (2021) reported one secondary outcome of the CORDIOPREV study (Coronary Diet Intervention with Olive Oil and Cardiovascular Prevention) which aimed to compare two healthy dietary patterns and their effects on the heart [84]. In a randomized controlled trial, 939 participants completed the Intima-media thickness of both common carotid arteries (IMT-CC) evaluation at baseline and after 5 and 7 years besides the carotid plaque number and height analysis. Participants were randomly divided into two groups: Mediterranean diet (35% fat, 22% MUFA, <50% carbohydrates) and low-fat diet (28% fat, 12% MUFA, >55% carbohydrates). The intake of EVOO rich MedDiet was linked to reduced atherosclerosis progression as opposed to a low-fat diet. The MedDiet lowered the carotid plaque max height and IMT-CC, noting that the latter was reduced at 5 years and maintained at 7 years, while no changes were observed after consuming the low-fat diet, supporting the MedDiet’s advantages in terms of secondary cardiovascular prevention.

Further trials, conducted within the framework of the CORDIOPREV study, have also supported the advantages of the MedDiet rich in EVOO as a secondary CVD prevention. This diet may have a preservation role for kidney function and a reduction in estimated glomerular filtration rate decrease in coronary heart disease patients with T2D, in comparison to the low-fat diet rich in complex carbohydrates [86].

Marrone et al. (2022) also studied the possible cardioprotective effects of EVOO rich in phenolic compounds in 40 chronic kidney disease patients under conservative therapy for the in vivo clinical testing by consuming 40 mL/day of raw EVOO for 9 weeks [89]. At the end of the study, inflammatory parameters, CIMT, and oxidative stress biomarkers decreased while the lipid and purine metabolism, atherogenic indices, and body compositions were enhanced [89].

The previously mentioned studies have experimented the benefits of OO on health, nevertheless, it is also important to compare the effects of MUFA, in the form of OO, and PUFA. Wu et al. (2022) explored this difference among middle-aged and elderly Chinese women at high cardiovascular risk, in a 3-month, randomized, controlled-feeding trial [85]. Ninety participants were randomized into 3 groups: diets using n-6 PUFA-rich soybean oil, MUFA-rich OO, and MUFA-rich camellia seed oil as cooking oils and as part of the traditional Chinese eating habits, consuming only the food given for lunch and dinner while refraining from taking edible oils for breakfast. No significant difference in body weight change among the three groups was noted. However, HDL-C had a minimal increase in the OO group and AST was more decreased in the camellia seed oil in comparison to the soybean oil. The study showed that MUFA-rich OO and camellia seed oil were more beneficial on the cardiometabolic profiles as compared to the n-6 PUFA-rich soybean oil.

A crossover, randomized trial, studied the cardiovascular health-related effects of isoenergetic included ghee or OO for 4 weeks in 30 healthy participants. The diet that included ghee increased the fasting plasma Apo-B and non-HDL cholesterol. Despite the nonsignificant differences between the two groups on LDL-C, this study emphasizes the recommendation of replacing SFA with unsaturated fats to decrease the risk of CVD [87].

Prater et al. (2022) conducted a randomized trial aimed to examine the blood lipid responses in 43 hypercholesterolemic adults after consuming enriched cottonseed oil (rich in PUFA) or OO (rich in MUFA) diets for 8 weeks [88]. It was concluded that the cottonseed oil was more effective in improving the fasting and postprandial blood lipids and postprandial glycemia in hypercholesterolemic adults. However, it is important to note that this study was a partial outpatient feeding intervention. The meals and snacks provided consisted of 60% of daily energy needs of which 30% came from cottonseed oil or OO. Additionally, pre- and post-diet interventions consisted of a high SFA meal (35% of total energy needs; 70% of energy from fat). This study did not use an EVOO, but a standard OO which did not include polyphenols’ bioactive properties known to lower the risk for cardiovascular diseases.

### 3.3. Olive Oil as an Antioxidant Vehicle

Many chronic diseases are a consequence of oxidative stress and inflammation triggered by increased oxidative stress. Accumulating evidence highlights the protective role that OO may play against such mechanisms and many studies described in Table 3 support the antioxidant and anti-inflammatory beneficial effects of OO in humans [65,91,92,93,94,95,96,97,98,99,100]. Many of these benefits are closely related to the content in PC of the OO consumed; in fact, most of the clinical trials performed over this period use EVOO, naturally rich in PC. Nonetheless, other bioactive compounds found in OO have also been implicated and their effect studied, such as triterpenes [97].

For example, the consumption of EVOO rich in PC improved metabolic control and circulating inflammatory adipokines profile in overweight T2D patients, by significantly reducing fasting plasma glucose, glycated hemoglobin (HbA1c), BMI, and body weight as well as reducing serum levels of AST, ALT, and visfatin [91]. Similarly, a case study with only T2D female patients, revealed thar OO consumption significantly reduced C-reactive protein (CRP) levels, thus reducing inflammation and oxidative stress which could improve diabetes complications [92].

In terms of enzymatic activity modulation, it was shown that in metabolic syndrome patients, from the PREDIMED cohort, a MedDiet supplemented with EVOO significantly decreased xanthine oxidase (an enzymatic source of reactive oxygen species (ROS)) activity and increased superoxide dismutase and catalase activities, which are key enzymes in the cell’s antioxidant system [93]. Within the same alignment, Luisi et al. [95] found that myeloperoxidase and 8-hydroxy-2-deoxyguanosine (markers of inflammation and oxidative stress), TNF-α, and IL-6 (proinflammatory cytokines) were significantly decreased in both normal weight and overweight/obese subjects who used high-quality EVOO (HQ-EVOO containing 365 mg/kg PC of which 2.65 mg/kg tyrosol and 2.37 mg/kg hydroxytyrosol) as the only cooking and dressing fat within a typical Mediterranean diet for 3 months [95].

With a special focus on the impact of PC, the more recent OLIVAUS double-blind crossover trial was conducted on 50 Australian individuals who were randomly divided and asked to consume 60mL/day extra-virgin high polyphenol OO or low polyphenol OO for a period of three weeks. Individuals were crossed over to the alternative treatment after a 2-week wash-out period. Although the results did not support significant differences between the two treatment groups the intake of high polyphenol (320 mg/kg) OO proved the antioxidant and anti-inflammatory effects of the OO which were more visible in those individuals with high cardiometabolic risk—there was a significant within-group reduction in plasma ox-LDL and CRP and an increase in plasma total antioxidant capacity (TAC) [94].

Longhi et al. (2021) studied, in an RCT, the effects of some nutritional interventions on the inflammatory biomarkers of 149 severely obese participants, aged between 18 and 65 years old, which were randomly separated into three groups: 52 mL/day of EVOO, DieTBra, and DieTBra plus 52 mL/day of EVOO [96]. DieTBra + EVOO was able to decrease significantly the total leukocytes and lymphocyte-to-monocyte ratio (LMR). DieTBra alone showed a minimal decrease in the neutrophil-to-lymphocyte ratio. EVOO and DieTBra interventions decreased CRP. It was also noted that the total leukocytes and LMR were similarly reduced in all groups.

More recently, other bioactive molecules found in OO have been implicated in the antioxidant potential of OO, namely OO triterpenes, which have been associated with decreasing DNA oxidation and plasma inflammatory biomarkers. In order to provide a better understanding of the impact of triterpenes in OO on antioxidant stress biomarkers Sanchez-Rodriguez and co-authors performed the NUTRAOLEUM trial with 51 healthy adults. The results revealed that OO enriched with triterpenic acids lowered urinary 8-hydroxy-2′-deoxyguanosine, plasma IL-8, and TNF-α concentrations, compared to the OO with less triterpenic acids [97].

Researchers have also looked into other antioxidant stress biomarkers to try to pinpoint OO role in management of associated diseases. For example, Loffredo et al. (2021) used soluble Nox2-derived peptide (sNox2-dp) levels to show the antioxidant potential of EVOO-enriched chocolate consumption by T2D patients—the levels were substantially decreased [65].

In a different setting, individuals with IFG who received a meal supplemented with 10 g of EVOO exhibited a noticeable reduction in post-prandial lipopolysaccharides (LPS). Patients who did not receive EVOO, however, had significantly higher levels of serum oxidative indicators such as sNox2-dp, LPS, Apo B-48, and ox-LDL. The decrease in LPS indicates that EVOO may affect chylomicron production and consequently LPS translocation from the gut, mitigating the oxidative stress-related inflammation characteristic of post-prandial phase [98].

A clinical intervention trial in trained male cyclists demonstrated that supplementation with 6 g/day EVOO decreased the methylation of the gene encoding IL-6 and the expression of DNA methyltransferase-1 (DNMT1). Therefore, there is a potential for dietary supplementation with EVOO to prevent the exercise-induced inflammation via the modulation of DNA methylation [99].

On the other hand, a recent trial, performed on patients with depression who consumed 25 mL/day of EVOO and SFO, showed no results on inflammation or oxidative stress biomarkers. Nonetheless, EVOO significantly decreased waist circumference and significantly increased HDL-C without the need for administration of a low-calorie diet [100].

These findings demonstrate the preventive antioxidant and anti-inflammatory potential effects of OO on healthy individuals, especially patients which are mostly in need for primary prevention initiatives.

### 3.4. Olive Oil Effects on Mental Health and Neurodegenerative Diseases

Pharmacological and psychological interventions are common strategies used to treat and prevent the risk of mental and neurodegenerative diseases. Besides these, other interventions based on lifestyle changes, like diet, physical activity, or alcohol and drug limitations, can also help the treatment of these diseases [101]. Diet has emerged as an important modifiable risk factor, and several studies have investigated the role of OO on cognitive performances and psychological health, suggesting it may have a neuroprotective effect [102]. Table 4 describes the main clinical studies that have been performed recently with OO and its impact on mental health and neurodegenerative protection [103,104,105,106,107,108,109,110].

In their one-year study on the impact of the MedDiet, Mazza et al. (2018) showed that the inclusion of a low dose (20–30 g/day) of EVOO resulted in a high improvement of cognitive functions among elderly individuals (aged ≥ 65 years), suggesting that a change in the quality of vegetable oils would improve cognitive functions better than the quantity, since EVOO (280 mg/kg PC; <0.8% acidity) is the best quality oil [103]. Still within dietary patterns, but in the context of the DieTBra, an RCT that was performed with individuals with severe obesity revealed that the traditional DieTBra and EVOO, alone or in combination, resulted in a significant reduction of anxiety and depression symptoms in this specific population group [104].

Another study highlighted the potential beneficial effect of OO on the cognitive function of women with HIV, increasing their attention/concentration scores by 4.2 points [105].

Two studies were performed within the Greek population with people with mild cognitive impairment. Results showed that the consumption of high-phenolic early-harvest EVOO lead to a significant improvement in cognitive function [106], reduced the over excitation of information flow in spontaneous brain activity, and increased brain flexibility after 12 months [107].

Two other studies, by Tzekaki et al. (2021), suggested that EVOO could be considered as a therapeutic strategy against Alzheimer’s disease (AD). The administration of EVOO in mild cognitive impairment patients for 1 year resulted in an increase of the neuroprotective protein BMI1 and a decrease of p53, of AD biomarkers (tau, p-tau, Aβ1–40, Aβ1–42, and Aβ1–42/Aβ-40 ratio), and of fibrinolytic system factors (PAI-1, α2AP, and tPA), assumed to be involved in AD pathophysiology. Also, oxidative stress and inflammatory responses were reduced [108,109].

Kaddoumi et al. (2022) performed a randomized, controlled trial on 25 mildly cognitively impaired participants, where the daily consumption of 30 mL/day of EVOO (1200 mg/kg total phenols) for 6 months significantly ameliorated the clinical dementia rating and behavioral scores, enhanced the functional connectivity, and decreased the blood–brain barrier permeability. The group that consumed ROO over the same period, however, did not have changes in the blood–brain barrier permeability and brain connectivity. The clinical dementia rating and the functional brain activation to memory tasks in cortical regions related to cognition and perception were improved. It was concluded that both ROO and EVOO are beneficial, with the latter having additional effects due to its bioactive phenolic content (mainly oleocanthal and oleacein) [110].

All these studies imply that OO, a completely natural product, can be used in clinical protocols as complementary treatment of cognitive impairments, anxiety, and depression. These interventions can be applied on a large scale to deal with these mental health impairments, considering the lack of adverse effects and the relative easiness of OO inclusion in the dietary routine.

### 3.5. Olive Oil’s Effects on Gut Microbiota

Gut microbiota are a key factor in driving metabolic activities and is involved in the regulation of host immunity. Modification of gut microbiota composition is associated with increased risk of metabolic and immune disorders in humans [111]. High-fat and high-sugar diets may alter intestinal microbiota causing modifications responsible for some of the medical disorders observed in metabolic syndrome [112]. In the intestine, OO PC can interact with the gut microbiota population and potentially influence the oxidative status of intestinal barrier, inflammation, and the immune response of the host [113]. Despite the significant impact that dietary gut microbiota modulation may play, studies looking into the potential of OO specificity are still at early stages of development. The six clinical trials that have been performed over the period under scrutiny essentially supported the potential prebiotic effect that PC may play.

Within the previously discussed VOHF study (12 hypercholesterolemic adults: 7 men and 5 women, reporting TC > 200 mg/dL, age: 46–67 years old) it was shown that the ingestion of VOO (30 mL/day), enriched in a mixture of OO and thyme PC, decreased blood ox-LDL concentrations, and increased the numbers of bifidobacteria and the levels of protocatechuic acid, a phenolic microbial metabolite with antioxidant activities, thus contributing to a cardio-protective effect. The specific growth stimulation of bifidobacteria in the human gut suggested for the first time a potential prebiotic activity of such a PC-enriched VOO [81].

In their study concerning the MedDiet, already presented in the previous Section 3.3, Luisi et al. (2019) further demonstrated that enrichment with HQ-EVOO (40 g/day for 3 months) induced an increase in the numbers of lactic acid bacteria found in the gut microbiota profile, where their probio-active cellular substances could produce beneficial effects in the gastrointestinal tract [95]. Recall that decreased oxidative stress and inflammation parameters, and increased adiponectin and IL-10 concentrations were also observed, as previously discussed.

On a more specific note, considering a particular target population group, the sustained consumption of EVOO (50 mL/day for 12 weeks) by HIV (human immunodeficiency virus)-infected patients between 50 and 75 years of age has a beneficial effect associated with a significant increase in alpha-diversity of intestinal microbiota in males [114]. A decrease in total cholesterol was also observed.

Besides preventive modulation, EVOO may also be used in the treatment of gastrointestinal disorders. For example, Morvaridi et al. (2020) showed in their single-blind crossover study involving 32 ulcerative colitis patients aged 18–65 years old and a BMI between 18.5 and 35 kg/m^2^, that EVOO could have an important beneficial role in the treatment of ulcerative colitis, an immune-mediated disease, which causes inflammation in the gastrointestinal tract [115]. Indeed, after EVOO intake (50 mL/day), inflammatory markers (ESR and CRP) and gastrointestinal symptoms (bloating, constipation, fecal urgency, and incomplete defecation) were significantly reduced in ulcerative colitis patients.

On the other hand, in a double-blind RCT, the intake of EVOO (25 mL/day for 9 weeks), associated with energy-restricted normofat diets, increased intestinal permeability, but did not affect the diversity and relative abundance of intestinal bacteria in 19 overweight women; LPS concentrations remained unchanged [116].

Finally, an 8-week double-blinded RCT studied the effects of 30 g of two blended cooking oils (high in omega-3 alpha-linolenic acid and phytonutrients) versus ROO on the intestinal microbiota in 126 participants having borderline hypercholesterolemia. The highest concentration of omega-3 PUFA oil blend was linked to the elevation of alpha diversity showing more robust results. It is important to note that EVOO was not used in this study, thus obtaining better results with the other blends as they are higher in tocopherols and phytonutrients [117].

### 3.6. Olive Oil’s Effects on Other Health Outcomes

Besides the main target benefits, a limited number of studies have shown the beneficial effects of OO on other health outcomes, targeting bone health, respiratory diseases, xerostomia, telomere lengths, and hip pain. Many more studies will be required before a robust cause–effect relationship can be established. Nonetheless, given the objective of this review to unravel the most recent findings concerning OO in clinical trials and its impact on health outcomes, these will also be briefly described below.

The consumption of OO has been shown to be effective in reducing bone loss and osteoporosis. Two studies demonstrated that a higher intake of OO (>18.32 g/day) significantly increased the volumetric bone mineral density [118] and that the mean consumption of 56.5 g/day of OO reduced the risk of osteoporotic fractures [119] in a cohort of 523 Spanish women across a wide range of ages (23–81 years of age, mean age of 50 years) and in a middle-aged and elderly (55–80 years of age) Mediterranean population (870 individuals) from the PREDIMED trial, respectively. A Brazilian randomized controlled trial found that 52 mL/day of EVOO has positive effects on the bone health parameters of severely obese adults, increasing calcium levels (from 9.5 ± 0.5 at baseline to 9.8 ± 0.5 at the end of the intervention) [111].

In former studies, dietary fat intake has been associated with respiratory diseases, with conflicting results. However, these studies were mainly conducted among populations following a typical Western diet, which is rich in processed food, but not in high-quality fatty acids, such as OO. Few data are available for Mediterranean countries. Cazzoletti et al. (2019) performed a population multi-case study (GEIRD project) of 871 Italian adults aged 20–84 years old, and results showed that the high consumption of MUFA, oleic acid and OO decreased the risk of current asthma but not of rhinitis [120].

A randomized clinical trial with 60 elderly patients with drug-induced xerostomia (dry mouth) showed that the oral application of lycopene-enriched VOO in spray form (3x/day) significantly improved their symptoms [121].

As a nonpharmacological intervention, EVOO and DieTBra, alone or combined, were found to be beneficial in decreasing pain and pain intensity in various spots, like the hips, among 149 severely obese adults over a 12-week randomized, controlled, parallel clinical trial [122].

## 4. Discussion

Non-communicable diseases are among several lifestyle and age-associated pathological conditions, such as cancer, CVD, diabetes mellitus, and neurodegenerative diseases. These are long lasting and slowly progressive, representing the leading causes of death and disability. The prevalence of non-communicable diseases has been rapidly increasing, and there is a need to find new preventive approaches to reduce their risk and burden. Research has highlighted the importance of a healthy lifestyle, consisting of regular physical activity and a balanced diet. The MedDiet is an excellent model of healthy eating, and its main characteristic is OO usage.

The present review provides clinical evidence supporting the positive impact of OO consumption on human health. OO, particularly EVOO, was shown to be associated with antioxidant and anti-inflammatory effects, improvement in endothelial function and lipid profile, prevention of obesity, diabetes, cardiovascular, and neurodegenerative diseases, and modulation of the gut microbiota. In this context, this review is one of the first to assess the positive contribution of OO consumption, independently, or within the context of the MedDiet, on cognitive function and neuroprotection as well as on gut microbiota diversity. Furthermore, the selected clinical studies demonstrated the synergy between olive oil minor bioactive compounds (phenolic compounds and triterpenes including oleocanthal, oleuropein, and hydroxytyrosol) and olive oil lipids (predominant MUFA) in many cases.

It is important to note that the MedDiet is considered a healthy dietary and nutritional pattern, associated with a reduced risk of cardiovascular events [123]. Most importantly, the health benefits of the MedDiet have been attributed to its lipid profile, high monounsaturated fat, but also to OO antioxidant capacity, mostly represented by EVOO. Indeed, EVOO has gathered a significant body of evidence of its beneficial health effects, independently of its association with dietary pattern. In the PREDIMED study, which was included in some of the subsections of this review, it was shown that EVOO reduced the risk of cardiovascular events compared with participants not taking EVOO [56]. Interestingly, Violi et al. have shown that after Mediterranean meals a significant difference was observed between participants with vs. without EVOO, specifically in relation to LDL-C and its oxidized form, which were significantly increased in the non-EVOO group. It is well established that LDL-C is a risk factor for the development of CVD. Additionally, when a meal not containing EVOO was given, LDL-C, ox-LDL, and triacylglycerols increased significantly [123]. Furthermore, the NUTRAOLEUM RCT Study, for example, highlighted the EVOO triterpenes’ health benefits in addition to their bioavailability and disposition in EVOO [97]. Other independent studies provided additional benefits for cardiovascular protection including improved insulin sensitivity and reductions in inflammatory biomarkers.

The demonstrated antioxidant properties of EVOO also need to be highlighted, and these may play a significant protective role against immune-mediated inflammatory responses including neurodegenerative diseases as supported by several of the clinical studies reported herein. The positive impact of EVOO on hepatic steatosis reduction was also of significance. Lastly, but of relevance to the previous benefits, were the clinical studies showing an important contribution of EVOO to gut microbiota composition modulation and associated metabolic activity. Although further studies are required, such an impact may positively influence metabolic and cognitive health.

Indeed, due to its disease-preventing effects, OO is considered a functional food [124]. In 2011, the European Food Safety Authority approved a health claim for OO, stating “OO polyphenols contribute to the protection of blood lipids from oxidative stress”. The claim may be used only for OO which contains at least 5 mg of hydroxytyrosol and its derivatives (e.g., oleuropein complex and tyrosol) per 20 g of OO. To bear the claim, information shall be given to the consumer that beneficial effects are obtained with a daily intake of 20 g of OO [125].

Despite the growing evidence supporting OO health benefits, inconclusive or contradictory effects have been reported in some cases. Such observation can be attributed to differences in study designs, sample sizes, participant characteristics, OO types and its endorsed doses, time-length interventions, and follow-up periods, among others. Therefore, one of the most important recommendations of this review, corroborating that of many other authors, is of the need to harmonize study designs to enable better comparisons and extract conclusions.

Furthermore, aside from the many positive effects of OO on human health, we continue to lack information on the precise mechanisms involving these processes. Further studies are needed to shed light on the understanding of the molecular mechanisms, to establish compound–benefit relationships, to define the effective doses in humans, independent of the health status, and to reveal new therapeutic potentials.

Although not directly related to what concerns the trials per si but important from the OO perspective itself are strategies to enhance its functionality in terms of composition. Since PC were highlighted as some of the most effective bioactive compounds, together with the lipid fractions, a strategy for their increase in OO may be via polyphenol oxidase activity. These enzymes seem to have a beneficial role in forming the phenolic glycosides in the olives where tyrosol is converted to hydroxytyrosol via hydroxylation. The polyphenol oxidase genes subfamilies are tyrosinases and diphenolases. These genes should be studied to be used in olive breeding programs aimed at enhancing the functional properties of oils [126].

## 5. Concluding Remarks

In conclusion, scientific data showed that OO intake, as a part of the MedDiet or as a functional food on its own, plays a critical role in the human organism, improving health and reducing the risk of certain diseases. These benefits can be attributed to a synergistic effect of the polyphenol compounds with the high content of oleic acid. Although additional research is required, an accumulating body of clinical evidence provides support for the consumption of EVOO as a balanced dietary element contributing positively toward prevention and management of a variety of non-communicable diseases.

## Figures and Tables

**Table 1:** Studies examining various interventions with OO consumption and their beneficial outcomes on metabolic outcomes.

Study Design	Compound	Intervention	Outcomes/ Biomarkers	Results	Reference
Clinical trial on 12 healthy subjects (age: 27–31 years old) and 12 patients with metabolic syndrome (age: 32–38 years old), 12 men and 12 women	High-Polyphenol Extra-Virgin Olive Oil (HP-EVOO) Low-Polyphenol Extra-Virgin Olive Oil (LP-EVOO)	Acute ingestion of 50 mL of HP-EVOO or LP-EVOO, after 1-week washout period and overnight fasting	Gene and miR expression analysis	In healthy subjects, HP-EVOO improved glycaemia and insulin sensitivity, and modulated the transcription of genes and miR involved in metabolism, inflammation, and cancer, modifying to a less deleterious inflammatory phenotype; In healthy subjects and in patients with metabolic syndrome, LP-EVOO showed weaker effects	[48]
RCT, crossover on 17 overweight women, age: 20–50 years old, BMI: 25–29.9 kg/m^2^	OO	Two 6-week periods, separated by a 2-week washout period, to consume either a usual diet or an OO-rich diet	Omentin and adiponectin	OO-rich diet tended to increase omentin and adiponectin	[29]
Randomized, blind, parallel on 43 subjects with prediabetes, 25 men and 18 women	EVOO	12 weeks of isocaloric weight-maintaining diet containing MUFA (OO) or extra fiber or habitual food (control diet)	Hepatic fat, glucose tolerance, insulin action and secretion	Diet rich in MUFA from OO significantly decreased hepatic fat and improved both hepatic and total insulin sensitivity	[45]
Randomized, crossover on 30 patients with impaired fasting glucose, 17 men and 13 women, age: 45–70 years old	EVOO	Patients were randomized to receive a meal including or not 10 g of EVOO in a crossover design; there was an interval of at least 7 days between the two phases of the study	Post-prandial glucose, lipid profile, glucose, insulin, GLP-1, DPP4, TG, total cholesterol, HDL, Apo B-48	EVOO reduced glucose and DPP4, significantly increased insulin and GLP-1, and significantly decreased TG and Apo B-48; total cholesterol and HDL levels did not significantly change; EVOO improved post-prandial glucose and lipid profile with a mechanism probably related to incretin up-regulation	[35]
Randomized double blind, crossover on 13 healthy young men, BMI: 23–25 kg/m^2^ age: 22–24 years old	OO	On 4 occasions, each separated by 3–14 days, ingestion of a control drink (450 mL) or iso-volumetric drinks containing protein/carbohydrate/fat: (1) 14 g/28 g/12.4 g, (2) 70 g/28 g/12.4 g, (3) 70 g/0 g/0 g	Gastric emptying, glucose, insulin, ghrelin, CCK, GLP-1, total energy intake	The substitution of whey protein with carbohydrate (dextrose) and fat (OO) resulted in faster gastric emptying, reduced suppression of ghrelin, and less stimulation of CCK and GLP-1; meanwhile, the addition of carbohydrate and fat to whey protein did not further slow gastric emptying, suppress ghrelin, or increase CCK and GLP-1 responses	[31]
Unstratified case-cohort study within the PREDIMED study on 251 patients with T2D and 641 without T2D, age: 60–73 years old	EVOO	1 year of intervention, 3 groups: MedDiet + EVOO (4 tablespoons/day/person), MedDiet + nuts (30 g mixed nuts—walnuts, hazelnuts, and almonds), low-fat diet	Plasma levels of amino acids	MedDiet + EVOO significantly lowered the levels of branched-chain amino acids (BCAAs)	[47]
RCT, single blind on 43 patients with NAFLD, 26 men and 17 women, BMI: 29.7 ± 0.58 kg/m^2^ age: 36–56 years old	VOO	12 weeks, 2 groups consuming a hypocaloric diet: (1) enriched with OO, or (2) with normal fat	ALT, AST, liver steatosis severity	Diet containing OO significantly decreased weight and ALT and AST levels, but the severity of liver steatosis did not change significantly during the study.	[34]
Randomized, double blind, crossover on 100 healthy adults, 47 men and 53 women, mean age: 40 years old, BMI: 18.5–24.9 kg/m^2^	OO	After 2-week run-in period, ingestion of 48 g/day of palm olein or OO during two phases of 5-week intervention period with a 2-week washout period between them	Anthropometric data and lipid profile	Palm olein and OO had no significantly different effect on body fatness or blood lipids	[40]
Randomized, single blind, crossover on 13 overweight patients with T2D, 8 men and 5 women, BMI: 30 ± 4.3 kg/m^2^ age: 47–75 years old	EVOO	On 3 different days, separated by 2–10 days, in random order, acute intake of either: (a) 200 g carrot, (b) 19 g EVOO + 200 g carrot, or (c) 10.7 g C4 dietary oil + 200 g carrot	Secretion of gut and pancreatic hormones	Both EVOO and C4-dietary oil resulted in greater secretion of GLP-1 and GIP	[41]
RCT, parallel, not blind on 94 healthy subjects, 31 men and 63 women, age: 50–75 years old	EVOO	4 weeks consumption of 50 g/day of one of 3 different dietary fats: EVOO, coconut oil or butter	Blood lipid profile, weight, fat distribution, and metabolic markers	Butter significantly increased LDL, TC/HDL ratio and non-HDL; coconut oil significantly increased HDL; there were no significant differences on weight, fat distribution and metabolic markers among any of the three dietary fats	[49]
RCT, parallel, multicenter, from the PREDIMED study on 7447 high CVD risk patients, >90% overweight or obese, 4282 women and 3165 men, age: 55–80 years old	EVOO	5 years of intervention, 3 groups: MedDiet + EVOO (50 mL/day), MedDiet + nuts (30 g/day), low-fat diet	Bodyweight and waist circumference	MedDiet enriched with EVOO or nuts showed small reductions in weight and lesser increases in waist circumference	[25]
Randomized, crossover on 67 healthy adults, 33 men and 34 women, BMI: 19.2–22.6 kg/m^2^ age: 21–25 years old	EVOO	18-weeks: first phase was a 2-week run-in period, followed by 3 phases of 4-week experimental period with a 2-week washout period between them; 3 groups of subjects consumed alternately a Chinese diet containing one of the 3 fats: EVOO, palm olein or cocoa butter	Lipid profile and LDL subfractions	Palm olein significantly lowered serum TG concentrations than EVOO; All the other lipid indices and LDL subfractions showed no significant differences amongst the three test fats	[50]
RCT, multicenter, parallel-group, subgroup analysis of PREDIMED trial on 100 men and women at high CVD risk, age: 55–80 years old	EVOO	3-year follow up, 3 groups: Med Diet + EVOO 60 mL/day, Med Diet + 30 g/day nuts, Low-fat diet (control group)	Hepatic steatosis	Med Diet + EVOO is associated with a lower prevalence of hepatic steatosis	[32]
RCT, crossover on 11 patients with T1D, 5 men and 6 women, age: 32–50 years old	EVOO	Consumption, alternating at 1-week intervals of one of 3 experimental meals, with different amount and quality of fat: 37 g EVOO (high-MU fat), 43 g butter (high-saturated fat), and 8 g (low fat)	Glucose, gastric emptying rate, GLP-1, GIP, glucagon, lipids	EVOO reduced glucose and significantly increased gastric emptying rate, GLP-1 and TG; GIP and glucagon were not significantly different between EVOO and butter	[36]
RCT, parallel, double-blind on 66 adults with NAFLD, BMI: 25 kg/m^2^	OO	12 weeks, 2 groups consuming: 20 g/day OO or 20 g/day SFO, both combined with a hypocaloric diet	Fatty liver grade, liver enzymes, anthropometric data, blood pressure, serum lipid profile, glucose, insulin, malondialdehyde (MDA), TAC, IL-6	OO intake lessened fatty liver grade and reduced body-fat percentage but did not affect liver enzymes and cardiometabolic risk factors; OO and SFO reduced weight, waist circumference, blood pressure, and serum aminotransferases	[33]
Randomized, single blind, crossover on 10 patients with T2D, 9 women and 1 man, BMI: 20–30 kg/m^2^ age: 30–60 years old	EVOO	EVOO or rice bran oil (15 mL/day) was administered for 4 weeks, followed by a 2-week washout period and a crossover for another 4 weeks	Glycemic control and lipid profiles	EVOO or rice bran oil significantly decreased only the HDL levels	[39]
RCT, parallel, double-blind, multicentre, PREDIABOLE study on 176 prediabetic subjects (with IFG and impaired glucose tolerance (IGT)), BMI: 25–39.9 kg/m^2^ age: 30–80 years old	OA-enriched OO non-enriched OO	55 mL/day of OA-enriched OO or non-enriched OO during 25–30 months	New-onset T2D incidence	The intake of OA-enriched OO substantially reduced the risk of developing T2D	[44]
RCT, parallel, multicenter, subgroup of PREDIMED study on 3230 patients with T2D, without treatment, 1552 men and 1678 women, age: 61–74 years old	EVOO	5 years of intervention, 3 groups: MedDiet + EVOO (1 L/week), MedDiet + nuts (30 g/day), low-fat diet	Need for glucose-lowering medications and for insulin treatment	MedDiet + EVOO significantly decreased the need for glucose-lowering medications; MedDiet, with EVOO or nuts, did not result in a lower need for insulin treatment	[46]
Experimental in 23 subjects with metabolic syndrome and hepatic steatosis, 15 men and 8 women, age: 49–71 years old	EVOO	EVOO with high oleocanthal concentration was given (32 g/day) for 2 months	Anthropometric data, metabolic parameters, hepatic steatosis, abdominal fat distribution, and pro- and anti-inflammatory cytokines	Oleocanthal-enriched EVOO significantly reduced body weight, waist circumference, body mass index, alanine transaminase, hepatic steatosis, pro-inflammatory cytokines (IL-6, IL-17A, TNF-α, IL-1B), while significantly increased anti-inflammatory cytokine (IL-10)	[24]
Cross-sectional observational study on 200 healthy adults, age: 20–30 years old	OO	Participants were divided in 2 groups regarding their OO consumption: low < 12.5 g/day, and high ≥ 12.5 g/day	BMI and waist circumference	OO consumption was not associated with increasing body mass index and waist circumference	[26]
RCT, parallel, blind on 111 adults with severe obesity, BMI ≥ 35 kg/m^2^ age: 18–64 years old	EVOO	12 weeks of intervention, 3 groups: DieTBra, EVOO (52 mL/day) and DieTBra + EVOO (52 mL/day)	Body composition and sarcopenia indicators	DieTBra + EVOO significantly reduced body weight and reduced total body fat; DieTBra significantly reduced body weight, and total body fat, and significantly improved walking speed and handgrip strength (sarcopenia indicators); EVOO alone did not improve any of the outcomes	[27]
Exploratory randomized crossover on 20 10 RYGB-operated patients and 10 controls	EVOO	Ingestion during 3 days, on separated occasions and in randomized order, of different triacylglycerol formulations: (1) 20 mL EVOO, (2) 13.8 mL C8-dietary oil, and (3) 10.7 mL tricaprylin	Enteroendocrine secretions, glucose, lipid, and bile acid metabolism	EVOO was significantly more effective in stimulating enteroendocrine secretion in RYGB-operated patients and controls, and gut hormone release was greater in RYGB-operated patients	[30]
RCT, crossover on 13 patients with T2D, 6 men and 7 women, age: 53–63 years old, BMI under the overweight or obese class	EVOO	Participants received a meal with or without EVOO followed by a 1-week washout period, after which they were given the other intervention	Postprandial blood glucose	Meals with EVOO increased the postprandial blood glucose, providing no additional benefit	[37]
RCT single blind, crossover on 25 patients with T2D and 20 healthy subjects, 22 men and 23 women, age: 27–77 year old	EVOO	One single intake of 40 g of oleuropein-enriched chocolate (addition of EVOO to enable final concentration of 4 mg% oleuropein) or 40 g of control chocolate spread. After 10 days washout phase, participants crossed over to take the opposite chocolate	Glucose, insulin	EVOO, as a source of oleuropein, is associated with a modest increase or no change of glycemia in T2D and healthy subjects, respectively	[42]
RCT, double blind in 72 women with polycystic ovarian syndrome, age: 18–45 years old	OO	3 groups receiving 25 g/day of OO, CO or SFO for 10 weeks	Lipid profile and fatty liver severity	OO consumption resulted in no significant reduction in lipid profile; OO and CO significantly decreased fatty liver grade and HOMA-IR	[23]
Short-term, open clinical trial, proof-of-concept study on 41 adults, lean (BMI 18.5–24.9 kg/m^2^) and obese/overweight (BMI 25–35 kg/m^2^), age: 25–40 years old	EVOO	Participants were encouraged to increase their usual OO consumption by replacing their habitual vegetable oil for OO during 4 weeks	Brown adipose tissue	EVOO ingestion leads to increased brown adipose tissue activity by significant increase in leptin, secretin, FGF21 and 12, 13 di-HOME in lean but not in overweight/obese volunteers	[28]
RCT, double blind, crossover on 20 healthy normal-weight subjects, 10 men and 10 women, age: 23–25 years old	EVOO	2-weeks consumption of 100 g/day chocolate spread enriched with either EVOO or palm oil, followed by 1 week of washout period and another 2 weeks of the opposite treatment	Ceramides concentration, glucose and lipid metabolism, inflammatory markers, appetite regulation	EVOO-enriched chocolate spread consumption led to decreased circulating harmful sphingolipids, HOMA-IR and plasma insulin; no major significant changes in TC, TG, HDL, inflammatory markers, and appetite regulation were observed between the groups	[43]
RCT, parallel, within the PREDIMED study on 150 subjects free of T2D, BMI: 27–35 kg/m^2^	EVOO	1 year of intervention, 3 groups: MedDiet + EVOO, MedDiet + nuts, low-fat diet	Exosomal non-coding RNAs (Ribonucleic acid) (Long Non-Coding RNAs (lncRNAs), messenger RNA (mRNA) and miRs) modulation	MedDiet + nuts and MedDiet + EVOO modulated exosomal RNA content, with the former affecting a higher number of miR	[51]
RCT in 40 adults aged 18–64 years with T2D and class II/III obesity	EVOO	2 groups receiving EVOO or EVOO+DieTBra for 12 weeks	Glycemic parameters, inflammatory markers, BMI, and weight	DieTBra significantly reduced fasting insulin levels and decreased BMI, weight, serum levels of inflammatory cytokines, IL-1α and adiponectin and increased TNF-α showing its role in ameliorating inflammatory profiles and fasting insulin levels.	[38]

(12, 13 Di-HOME: 12,13-Dihydroxy-9Z-Octadecenoic Acid; ALT: Alanine Aminotransferase; Apo B-48: Apolipoprotein B-48; AST: Aspartate Aminotransferase; BCAAs: Branched-Chain Amino Acids; BMI: Body Mass Index; CCK: Cholecystokinin; CO: Canola Oil; CVD: Cardiovascular Disease; DieTBra: Traditional Brazilian Diet; DPP4: Dipeptidyl-Peptidase-4; EVOO: Extra-Virgin Olive Oil; FGF21: Fibroblast Growth Factor 21; GIP: Glucose-Dependent Insulinotropic Polypeptide; GLP-1: Glucagon-Like Peptide-1; HDL or HDL-C: High-Density Lipoprotein-Cholesterol; HOMA-IR: Homeostatic Model Assessment of Insulin Resistance; HPOO: High Polyphenol Extra-Virgin Olive Oil; IFG: Impaired Fasting Glucose; IGT: Impaired Glucose Tolerance; IL: Interleukin; LDL or LDL-C: Low-Density Lipoprotein-Cholesterol; lncRNAs: Long Non-Coding RNAs; LP-EVOO: Low-Polyphenol Extra-Virgin Olive Oil; MDA: Malondialdehyde; MedDiet: Mediterranean Diet; miR: MicroRNAs; mRNA: messenger RNA; NAFLD: Nonalcoholic Fatty Liver Disease; OA: Oleanolic Acid; OO: Olive Oil; RCT: Randomized Control Trial; RNA: Ribonucleic acid; RYGB: Roux-en-Y Gastric Bypass; SFO: Sunflower Oil; T2D: Type 2 Diabetes; TAC: Total Antioxidant Capacity; TC: Total Cholesterol; TG: Triacylglycerols; TNF-α: Tumor Necrosis Factor Alpha; VOO: Virgin Olive Oil).

**Table 2:** Studies examining various interventions with OO consumption and their beneficial outcomes on cardiovascular health.

Study Design	Compound	Intervention	Outcomes/ Biomarkers	Results	Reference
RCT, double-blind, crossover, subsample from EUROLIVE study on 18 healthy men, age: 20–60 years old	EVOO ROO	Ingestion of 25 mL/day f OO (366 mg/kg phenolic compounds) for 3 weeks, preceded by 2-week washout periods	Anthropometric and blood pressure measurements, TC, LDL, HDL, TG, glucose, blood pressure-related gene expression analysis	EVOO decreased systolic blood pressure, maintained diastolic blood pressure, and decreased ACE, NR1H2 and IL8RA gene expression	[59]
RCT, double blind, parallel on 41 women overweight and obese age: 26–28 years old	EVOO	9 weeks, 25 mL/day intake of one of the tested oils, EVOO or soybean oil, associated with energy-restricted normofat diets	Anthropometric, body composition and blood pressure measurements; metabolic biomarkers	EVOO consumption reduced body fat, diastolic blood pressure, alkaline phosphatase; increased serum creatinine; and tended to reduce IL-1β concentrations	[60]
RCT, parallel, multicenter, PREDIMED study on 7403 high CVD risk patients, age: 55–80 years old	EVOO	5 years of intervention, 3 groups: MedDiet + EVOO (1 L/week), MedDiet + nuts (210 g/week), low-fat diet	Heart failure incidence	MedDiet with EVOO and MedDiet with nuts showed no significant effect on lowering heart failure incidence	[73]
Prospective, case-cohort, nested in the PREDIMED study on 980 high CVD risk patients	EVOO	5 years of intervention, 3 groups: MedDiet + EVOO, MedDiet + nuts, low-fat diet	Plasma ceramides concentration	MedDiet enriched with EVOO or nuts showed the potential to mitigate the deleterious effects of elevated plasma ceramide concentration on CVD risk	[74]
RCT, parallel, multicenter, substudy of PREDIMED on 90 high CVD risk women, age: 60–80 years old	EVOO	1 year of intervention, 3 groups: MedDiet + EVOO (52 g/day), MedDiet + nuts (30 g/day), low-fat diet	Endothelial markers involved in blood pressure control	MedDiet with EVOO or nuts reduced blood pressure values	[62]
RCT, parallel, multicenter, subsample of PREDIMED study on 210 high CVD risk patients, age: 58–73 years old	VOO	1 year of intervention, 3 groups: MedDiet + VOO (1 L/week), MedDiet + nuts (210 g/week), low-fat diet	LDL atherogenic traits: resistance against oxidation, particle size, composition, cytotoxicity	MedDiet + VOO decreased LDL atherogenicity by increasing LDL resistance against oxidation, LDL particle size and composition (cholesterol-rich), and decreasing LDL oxidative modifications and particles cytotoxicity	[75]
RCT, parallel, multicenter, subsample of PREDIMED study on 296 high CVD risk patients, age: 59–72 years old	VOO	1 year of intervention, 3 groups: MedDiet + VOO (1 L/week), MedDiet + nuts (210 g/week), low-fat diet	HDL functionality	MedDiet, especially when enriched with VOO, improved HDL atheroprotective functions; both MedDiet increased cholesterol efflux capacity; MedDiet + VOO decreased cholesteryl ester transfer protein activity and increased HDL ability to esterify cholesterol, paraoxonase-1 arylesterase activity, and HDL vasodilatory capacity; the 3 diets increased the percentage of large HDL particles	[76]
RCT, single-center, placebo study on 60 postmenopausal women, age: 50–61 years old	EVOO	1 year of oral supplementation with placebo or EVOO enriched with vitamins D3, K1 and B6 20 mL/day	Platelet membrane fluidity, Na^+^/K^+^-ATPase activity, serum nitric oxide (NO), and peroxynitrite	EVOO enriched with vitamins decreased platelet membrane anisotropy, NO and peroxynitrite, and increased Na^+^/K^+^-ATPase activity	[68]
RCT, double-blind, crossover, post hoc analyses from VOHF study on 33 hypercholesterolemics adults, 19 men and 14 women, TC > 200 mg/dL, age: 35–80 years old	VOO FVOO FVOOT	The different OO will be sequentially ingested (30 mL/day) during three periods of 3 weeks, preceded by 2-week washout periods	HDL composition, fluidity, oxidation, size and cholesterol efflux capacity	VOO ingestion increased HDL fluidity and apolipoprotein A-I concentration in HDL, and decreased HDL oxidative status, which are main determinants for cholesterol efflux capacity enhancement	[80]
Case-cohort design, subcohort of PREDIMED study on 983 high CVD risk patients, age: 61–76 years old	EVOO	1 year of intervention, 3 groups: MedDiet + EVOO (50 g/day), MedDiet + nuts (30 g/day), low-fat diet	Lipid species	Although the MedDiet interventions, supplemented with EVOO or nuts, induced some significant changes in the lipidome, they were not significantly associated with subsequent CVD risk	[77]
RCT, double-blind, crossover, subsample from VOHF study on 12 hypercholesterolemic adults, 7 men and 5 women, TC > 200 mg/dL, age: 46–67 years old	VOO FVOO FVOOT	The different OO will be sequentially ingested (30 mL/day) during three periods of 3 weeks, preceded by 2-week washout periods	Blood lipids, faecal quantitative changes in microbial populations, short chain fatty acids, cholesterol microbial metabolites, bile acids, and phenolic metabolites	FVOOT decreased ox-LDL, increased bifidobacteria numbers, and increased protocatechuic acid levels	[81]
RCT, double-blind, crossover, VOHF study on 33 hypercholesterolemics adults, 19 men and 14 women, TC > 200 mg/dL, age: 35–80 years old	VOO FVOO FVOOT	The different OO were sequentially ingested (25 mL/day) during three periods of 3 weeks, preceded by 2-week washout periods	HDL fatty acids, HDL antioxidant content, HDL monolayer fluidity, HDL cholesterol efflux capacity	The FVOO and FVOOT increased HDL antioxidant content, but α-tocopherol was only augmented after FVOOT	[82]
RCT, prospective on 160 patients with T2D, 118 men and 42 women, age: 40–60 years old	OO	Diet without or with 1.1 mL of OO + 500 mg of garlic powder for 3 months	Serum cholesterol and serum TG	Combination of OO with garlic powder significantly normalized the cholesterol and TG levels	[55]
RCT double-blind, crossover, NUTRAOLEOUM Study on 51 healthy adults	VOO OVOO Functional Olive Oil (FOO)	VOO (124 ppm PC, 86 ppm triterpenes), OVOO (490 ppm PC, 86 ppm triterpenes) and FOO (487 ppm PC and 389 ppm triterpenes) all at (30 mL/day) were sequentially administered over three periods of 3 weeks preceded by 2-week washout periods	Metabolic syndrome and endothelial function biomarkers	VOO, OVOO, and FOO reduced the plasma endothelin-1 levels; no effect of triterpenes was observed.	[63]
RCT, parallel, multicenter, subsample of PREDIMED study on 7447 high CVD risk patients, 4282 women and 3165 men, age: 55–80 years old	EVOO	5 years of intervention, 3 groups: MedDiet + EVOO (50 mL/day), MedDiet + nuts (30 g/day), low-fat diet	CVD incidence	MedDiet supplemented with EVOO or nuts decreased the incidence of major cardiovascular events, including acute myocardial infarction, stroke and death for CVD	[72]
RCT, parallel, multicenter, subsample of PREDIMED study on 296 high CVD risk patients	VOO	1-year increases in the consumption of VOO (10 g/day), nuts (30 g/day), legumes (25 g/day), whole grains (25 g/day), and fish (25 g/day)	HDL functionality	Increases in the consumption of VOO, nuts, legumes, whole grains, and fish improved HDL functions; VOO increased cholesterol efflux capacity	[83]
Prospective, population-based study, ATTICA study on 2020 CVD-free adults, age: 18–89 years old	OO	10-year follow up; participants were classified into 3 groups: no use, mixed use, and exclusive use of OO	Fatal/non-fatal CVD incidence	Exclusive OO use decreased the risk of developing CVD	[52]
RCT, double-blind, multiarm parallel study on 86 healthy young adults, 43 men and 43 women, age: 18–30 years old	OO	12 weeks of 3 g/day supplementation of OO, eicosapentaenoic acid or docosahexaenoic acid	Resting hemodynamics and muscle sympathetic nerve activity	OO supplementation reduced resting systolic and diastolic blood pressure and reduced muscle sympathetic nerve activity	[61]
RCT, crossover, double-blind study on 7 healthy males, active runners engaged in endurance activities (10–14 h/week), age: 28–36 years old, BMI: 23.1 ± 1.7 Kg/m^2^	EVOO	Three separate effort test sessions were carried out separated by 7-day interval. During each session, participants repeated the same test, but under different acute dietary supplementation in a randomized order: EVOO (25 mL), palm oil (25 mL), and placebo	Cardiorespiratory coordination and performance	Supplementation with EVOO increased cardiorespiratory coordination during a progressive walking test at moderate intensity, although it did not change performance	[71]
Follow-up study on 92,978 adults: 61,181 women and 31,797 men, Free of cancer, heart disease, and stroke	OO	24-year follow up; OO intake was categorized into 4 categories: (1) never or <1/month; (2) >0 to ≤4.5 g/day; (3) >4.5 to ≤7 g/day; and (4) >7g/day	CVD, coronary heart disease and stroke risk; inflammatory and lipid biomarkers	Higher OO intake was associated with lower risk of coronary heart disease and CVD; in a subset of participants, higher OO intake was associated with lower levels of circulating inflammatory biomarkers and a better lipid profile	[53]
RCT, parallel-arm, open label study on 48 patients with at least one classic CVD risk factor (hypertension, dyslipidemia, or diabetes), 44 men and 4 women, age: 51–64 years old	ROO	Ingestion of 25 mL/day of ROO or CO for 6 weeks	Plasma lipids, some selected inflammatory markers, lipoprotein-associated phospholipase A2 (Lp-PLA_2)_ levels	OO consumption significantly decreased IL-6 concentration	[54]
RCT, parallel, single-center study on 204 patients with stable coronary artery disease, age: 40–80 years old	EVOO	12 weeks, 3 groups: healthy diet, healthy diet + 30 mL/day EVOO, healthy diet + 30 g/day pecans	TG, TC, LDL, HDL, non-HDL, TC/HDL ratio, LDL/HDL ratio, HDL/TG ratio, atherogenic index	There were no significant differences in LDL levels after the consumption of a healthy diet supplemented with EVOO or pecans; supplementing the healthy diet with pecan nuts may improve other lipid profile markers	[57]
RCT, double-blind, preliminary study on 30 women with fibromyalgia, age: 44–60 years old	EVOO ROO	Ingestion of 50 mL/day of EVOO or ROO for 3 weeks	Thrombosis-related parameters, ESR, inflammatory markers, NO levels, lipid profile and cortisol levels	Consumption of EVOO decreased significantly red blood cell count, ESR and cortisol levels. Consumption of ROO significantly increased mean platelet volume and cortisol levels, and reduced platelet distribution width, neutrophil-to-lymphocyte ratio, ESR, and fibrinogen. No significant changes in the lipid profile, inflammatory markers and NO levels	[67]
RCT, postprandial, parallel, double-blind, subsample from VOHF study on 20 healthy participants, age: 22–60 years old	EVOO	Acute intake of 30 mL of the EVOO after 12 h of fasting	Plasma miR related to CVD	All EVOO, regardless of polyphenol content, decreased the levels of let-7e-5p; Low Phenolic Content Extra-Virgin Olive Oil (L-EVOO) and Medium Phenolic Content Extra-Virgin Olive Oil (M-EVOO) increased miR-17-92 cluster	[70]
Cross-sectional analysis of the PREDIMED study on 4330 high CVD risk patients, with an ankle-brachial pressure index (ABI) <1.4 and total energy intakes: 800–4000 Kcal/day for men, 500–3500 Kcal/day for women	EVOO VOO ROO VOO mixture Olive-pomace oil	Consumption of any category of OO and olive-pomace oil was assessed through a validated food-frequency questionnaire	ABI	VOO (EVOO and VOO) consumption was associated with a higher mean ABI	[78]
Quasi-experimental on 84 healthy men and women, age: 23–85 years old, divided into 2 groups: 28 young (23–45 years) and 56 elderly (65–85 years)	EVOO	Consumption of 25 mL/day of raw EVOO for 12 weeks	Blood pressure, TC, LDL, HDL, TG, glucose, CEC of HDL, HDL subclasses distribution	EVOO significantly decreased the CEC of the HDL of elderly healthy subjects (to a level comparable to that of young healthy subjects), and improved distribution of HDL subclasses (increasing large HDL and decreasing small HDL particles)	[56]
RCT, parallel and unicentricstudy in 149 patients with stable coronary artery disease, age: 40–80 years old	EVOO	12 weeks, 3 groups: healthy diet, healthy diet + 30 mL/day EVOO, healthy diet + 30 g/day pecans	Plasma fatty acids	There were no significant differences in plasma fatty acids after the consumption of a healthy diet supplemented with EVOO or pecans	[58]
RCT, double-blind, crossover study on 20 adults at risk of T2D, 10 men and 10 women, age: 25–75 years old	EVOO ROO	50 mL single dose administration of each of 2 treatments (EVOO—189 ppm phenolic compounds or ROO ≤ 20 ppm PC) in random sequence, with a 1-week washout between treatment assignments	Endothelial function and blood pressure	EVOO acutely improved endothelial function; no significant effects on systolic or diastolic blood pressure were observed	[64]
RCT single-blind, crossover study on 25 T2D patients, 12 men + 13 women, age: 61–77 years-old	EVOO	One single intake of 40 g of EVOO-enriched chocolate or 40 g of control chocolate spread. After a 10-day washout phase, participants crossed over to take the opposite chocolate	Endothelial function and oxidative stress	EVOO-enriched chocolate is associated with increased endothelial function (increasing the arterial brachial flow-mediated dilation) and reduction of oxidative stress	[65]
Exploratory crossover study on 10 patients with T1D and 6 healthy subjects, 13 men and 3 women, age: 20–36 year old	EVOO	Each participant received 2 types of high glycemic index meal: one enriched with EVOO and one with butter	Endothelial function, glucose and lipids measurements, and gastric emptying assessment	EVOO, added to a single high glycemic index meal, significantly increased the endothelial function by increasing the arterial brachial flow-mediated dilation	[66]
Prospective study on 63 patients with severe obesity, age: 24–40 years old, BMI: 44.1 ± 8.5 kg/m^2^	OO	OO intake was stratified into <1 time/week, 1–3 times/week, ≥4 times/week	Platelet activation with and without thrombin exposure	More frequent OO intake reduced thrombin-induced platelet activation	[69]
Prospective, randomized, single-blind, controlled trial in 1002 coronary heart disease patients analysis	EVOO	2 groups following a MedDiet or low-fat diet monitored at baseline and after 5 and 7 years.	IMT-CC, carotid plaque number and height	EVOO-rich MedDiet was linked to reduced atherosclerosis progression and lower carotid plaque_max_ height and IMT-CC while no changes were observed with the low-fat diet group, evidencing the MedDiet’s advantages as secondary CVD prevention.	[84]
Three-arm, randomized, controlled-feeding trial in 90 middle-aged and elderly Chinese women at high cardiovascular risk	OO	3 groups using n-6 PUFA-rich soybean oil, MUFA-rich olive oil, or MUFA-rich camellia seed oil as cooking oils within traditional Chinese eating habits for 3 months	Body weight, cardiovascular profiles, HDL, and AST	MUFA-rich OO and camellia seed oil were shown to be more beneficial on the cardiometabolic profiles as they had a role in increasing HDL-C and decreasing AST, respectively.	[85]
CORDIOPREV randomized controlled trial in 1002 coronary heart disease patients	EVOO	2 groups followed a MedDiet or a low-fat diet monitored at baseline and after 5 years	Kidney function by determination of serum creatinine-based estimated glomerular filtration rate	The advantages of the MedDiet rich in EVOO as a secondary CVD prevention was supported as it may have a preservation role for kidney function and a reduction in estimated glomerular filtration rate decrease in coronary heart disease patients with T2D. HDL-C had a minimal increase in the OO group and AST was decreased more in camellia seed oil in comparison to the soybean oil.	[86]
A crossover, randomized trial in 30 healthy participants	OO	2 groups consuming isoenergetic ghee or OO for 4 weeks	Fasting plasma apo-B, non-HDL-cholesterol, LDL-cholesterol, total cholesterol:HDL-cholesterol ratio	The diet that included ghee increased the fasting plasma Apo-B and non-HDL cholesterol. Despite the non-significant differences between the two groups on LDL-C, this study emphasizes the recommendation of replacing SFA with unsaturated fats to decrease the risk of CVD.	[87]
A randomized trial in 43 hypercholesterolemic adults	OO	2 groups consuming cottonseed oil or OO diets for 8 weeks	blood lipid responses	The partial outpatient feeding intervention concluded that cottonseed oil was more effective in improving the fasting and postprandial blood lipids and postprandial glycemia in hypercholesterolemic adults.	[88]
A case-control study nested in the PREDIMED study; 167 peripheral artery disease cases matched with 250 controls	EVOO	3 groups: MedDiet with supplementation of tree nuts, MedDiet with EVOO supplementation, or control (low-fat diet)	Plasma amino acids and risk of peripheral artery disease	MedDiet+EVOO group was protected against peripheral artery disease regardless of baseline threonine which can be an early biomarker of future disease incidences in high-risk CVD individuals.	[79]
Study performed on 40 chronic kidney disease patients under conservative therapy for the in vivo clinical testing	EVOO rich in phenolic compounds	Participants consumed 40 mL/day of raw EVOO for 9 weeks	Inflammatory parameters, oxidative stress biomarkers, lipid and purine metabolism, atherogenic indices	Inflammatory parameters, carotid intima-media thickness (CIMT), and oxidative stress biomarkers decreased while the lipid and purine metabolism, atherogenic indices, and body compositions were enhanced.	[89]

(ABI: Ankle-Brachial Pressure Index; ACE: angiotensin I-converting enzyme; Apo B: Apolipoprotein B; AST: Aspartate Aminotransferase; BMI: Body Mass Index; CEC: Cholesterol Efflux Capacity; CIMT: carotid intima-media thickness; CO: Canola Oil; CVD: Cardiovascular Disease; ESR: Erythrocyte Sedimentation Rate; EVOO: Extra-Virgin Olive Oil; FOO: Functional Olive Oil; FVOO: Phenol-Enriched VOO; FVOOT: Phenol-Enriched VOO and Thyme; HDL or HDL-C: High-Density Lipoprotein-Cholesterol; IL: Interleukin; IL8RA: Interleukin 8 receptor alpha; IMT-CC: Intima-Media Thickness of Both Common Carotid Arteries; LDL or LDL-C: Low-Density Lipoprotein-Cholesterol; L-EVOO: Low Phenolic Content Extra-Virgin Olive Oil; Lp-PLA2: Lipoprotein-Associated Phospholipase A2; MedDiet: Mediterranean Diet; M-EVOO: Medium Phenolic Content Extra-Virgin Olive Oil; miR: MicroRNAs; MUFA: Monounsaturated Fatty Acids; NO: Nitric Oxide; NR1H2: nuclear receptor subfamily 1, group H, member 2; OO: Olive Oil; OVOO: Ordinary Virgin Olive Oil; PC: Phenolic Compounds; PUFA: Polyunsaturated Fatty Acids; RCT: Randomized Control Trial; ROO: Refined Olive Oil; SFA: Saturated Fatty Acids; T1D: Type 1 Diabetes; T2D: Type 2 Diabetes; TC: Total Cholesterol; TG: Triacylglycerols; VOHF: VOO and HDL Functionality VOO: Virgin Olive Oil).

**Table 3:** Studies examining various interventions with OO consumption and their beneficial outcomes, consequence of OO’s antioxidant properties.

Study Design	Compound	Intervention	Outcomes/ Biomarkers	Results	Reference
RCT, parallel-group, multicenter, from the PREDIMED study on 75 metabolic syndrome patients, age: 55–80 years old	EVOO	5 years of intervention, 3 groups: MedDiet + EVOO (50 g/day), MedDiet + nuts (30 g/day), low-fat diet	Antioxidant capabilities and xanthine oxidase activity	MedDiet + EVOO and MedDiet + nuts decreased xanthine oxidase activity and increased superoxide dismutase and catalase levels and antioxidant activities	[93]
Experimental on 11 overweight and non-insulin treated T2D Caucasian patients, 7 men and 4 women, mean age: 64.63 ± 8.52 years old, diabetes duration ≤10 years	ROO HP-EVOO	8 weeks: intake of ROO (25 mL/day) for the first 4 weeks (wash-out period) followed by intake of HP-EVOO (25 mL/day) for the rest of 4 weeks	Anthropometric parameters, fasting plasma glycaemia, HbA1c, CRP, plasma lipid profile, liver function, and serum levels of TNF-α, adiponectin, IL-6, visfatin, apelin	HP-EVOO consumption reduced fasting glucose, HbA1c, BMI, body weight, AST, ALT, and visfatin	[91]
RCT, parallel group, single-center study on 77 T2D women, age: >50 years old; mean BMI 28 kg/m^2^	OO	8 weeks, 3 groups: balanced diet + 30 g/day of OO or CO or SFO	Height, weight, waist circumference, fasting blood sugar, serum insulin, CRP, MDA	CRP level was reduced significantly in OO and CO groups	[92]
Two-arm study on 18 overweight/obese subjects (BMI ≥ 25 kg/m^2^) + 18 normal weight controls (BMI 18.5–24.9 kg/m^2^)	HQ-EVOO	MedDiet enriched with 40 g/day of HQ-EVOO for 3 months	Lactic acid bacteria composition, oxidative stress, metabolic and inflammation parameters	MedDiet rich in HQ-EVOO increased lactic acid bacteria numbers, decreased oxidative stress and inflammation parameters, and increased adiponectin and IL-10 concentrations	[95]
RCT double-blind, crossover, NUTRAOLEOUM study on 51 healthy adults	VOO OVOO FOO	VOO, OVOO and FOO (30 mL/day) were sequentially administered over three periods of 3 weeks preceded by 2-week washout periods	Oxidative and inflammatory biomarkers.	Urinary 8-hidroxy-2′-deoxyguanosine, plasma IL-8 and TNF-α were lower after the intervention with the FOO than after the OVOO. IL-8 was lower after the intervention with FOO than after VOO intervention.	[97]
RCT, crossover on 30 patients with IFG, 17 men + 13 women, mean age: 58 years-old	EVOO	Taking a meal with or without 10 g of EVOO	LPS, Apo-B48, ox-LDL, sNox2-dp, plasma polyphenols	EVOO significantly decreased LPS, ox-LDL, sNox2-dp and plasma polyphenols	[98]
RCT, double-blind, crossover study on 8 male trained cyclists, age: 34–45 years-old	EVOO	Four-week supplementation of n-3 PUFA (5.7 g/day) or EVOO (6 g/day), followed by a four-week washout and crossover to the other supplement	Global and gene-specific (PPARGC1A, IL6 and TNF) DNA methylation, and DNMT1 mRNA expression	EVOO decreased the methylation of the gene encoding IL-6 and the expression of DNMT1	[99]
RCT double-blind, crossover, OLIVAUS study on 43 healthy Australian adults, age: 38.5 ± 13.9 years old, 66% females	High Polyphenol Extra-Virgin Olive Oil (HPOO—320 mg/kg PC) Low Polyphenol Extra-Virgin Olive Oil (LPOO—86 mg/kg PC)	Consumption of 60 mL/day of HPOO or LPOO for 3 weeks. Following a 2-week wash-out period, participants crossed-over to the alternate treatment	Anthropometric parameters, TAC, plasma oxLDL, CRP	After HPOO consumption it was observed a reduction in ox-LDL and CRP and an increase in TAC, although there were no significant differences between treatments	[94]
RCT single-blind, crossover study on 25 T2D patients, 12 men + 13 women, age: 61–77 years-old	EVOO	One single intake of 40 g of EVOO-enriched chocolate or 40 g of control chocolate spread. After a 10 days washout phase, participants crossed over to take the opposite chocolate	Endothelial function and oxidative stress	EVOO-enriched chocolate is associated with increased endothelial function and reduction of oxidative stress (decreasing sNox2-dp)	[65]
RCT double-blind study on 62 patients with major depression, age: 18–65 years-old	EVOO	52 days, during which participants consumed 25 mL/day of EVOO or SFO	Weight, BMI, waist circumference. TG, TC, LDL, HDL, Very Low-Density Lipoprotein Cholesterol (VLDL), MDA, CRP	EVOO significantly decreased waist circumference and significantly increased HDL	[100]
RCT in 149 severely obese individuals aged 18–65 years	EVOO	2 groups: nutritional EVOO and DieTBra+EVOO followed for 12 weeks	Inflammation profiles, neutrophil-to-lymphocyte ratio, LMR, leukocytes, and CRP	DieTBra+EVOO was able to significantly decrease the total leukocytes and LMR. DieTBra showed a minimal decrease in neutrophil-to-lymphocyte ratio. EVOO and DieTBra interventions decreased CRP. It was also noted that the total leukocytes and LMR were similarly reduced in all groups.	[96]

(ALT: Alanine Aminotransferase; Apo B-48: Apolipoprotein B-48; AST: Aspartate Aminotransferase; BMI: Body Mass Index; CO: Canola Oil; CRP: C Reactive Protein; DieTBra: Traditional Brazilian Diet; DNMT1: DNA Methyltransferase-1; EVOO: Extra-Virgin Olive Oil; FOO: Functional Olive Oil; Hba1c: Glycated Hemoglobin; HDL or HDL-C: High-Density Lipoprotein-Cholesterol; HP-EVOO: High-Polyphenol Extra-Virgin Olive Oil; HPOO: High Polyphenol Extra-Virgin Olive Oil; HQ-EVOO: High Quality EVOO; IFG: Impaired Fasting Glucose; IL: Interleukin; LDL or LDL-C: Low-Density Lipoprotein-Cholesterol; LMR: Lymphocyte-to-Monocyte Ratio; LP-EVOO: Low-Polyphenol Extra-Virgin Olive Oil; LPOO: Low Polyphenol Extra-Virgin Olive Oil; LPS: Lipopolysaccharides; MDA: Malondialdehyde; MedDiet: Mediterranean Diet; mRNA: messenger RNA; OO: Olive Oil; OVOO: Ordinary Virgin Olive Oil; Ox-LDL: Oxidized-LDL; PUFA: Polyunsaturated Fatty Acids; RCT: Randomized Control Trial; ROO: Refined Olive Oil; SFO: Sunflower Oil; sNox2-dp: Soluble NADPH oxidase 2-Derived Peptide; T2D: Type 2 Diabetes; TAC: Total Antioxidant Capacity; TC: Total Cholesterol; TG: Triacylglycerols; TNF-α: Tumor Necrosis Factor Alpha; VLDL: Very Low Density Lipoprotein Cholesterol; VOO: Virgin Olive Oil).

**Table 4:** Studies on various interventions with OO consumption and their beneficial outcomes on mental health and neurodegenerative diseases.

Study Design	Compound	Intervention	Outcomes/ Biomarkers	Results	Reference
RCT on 180 elderly individuals, aged ≥ 65 years	EVOO	1 year, 2 groups: MedDiet + EVOO (20–30 g/day) and control MedDiet	Cognitive functions	MedDiet + EVOO resulted in a higher improvement of cognitive functions, compared with MedDiet alone	[103]
RCT, parallel on 129 adults with severe obesity, age: 18–65 years old, BMI ≥ 35 kg/m^2^	EVOO	12 weeks of intervention, 3 groups: DieTBra, EVOO (52 mL/day) and DieTBra + EVOO (52 mL/day)	Anxiety and depression	DieTBra and EVOO, alone or in combination, resulted in a significant reduction of anxiety and depression symptoms	[104]
Cross-sectional analysis of 166 women aging with HIV	OO	Evaluation within 18 months, women were divided into those who reported using OO and those who did not	Cognitive performance	OO increased their attention/concentration scores	[105]
RCT, prospective, longitudinal, double-blind, MICOIL study on 60 patients with mild cognitive impairment, age: 60–80 years old	High-Phenolic Early Harvest Extra-Virgin Olive Oil (HP-EH-EVOO) Moderate-Phenolic Extra-Virgin Olive Oil (MP-EVOO)	1 year of intervention, 3 groups: HP-EH-EVOO (50 mL/day), MP-EVOO (50 mL/day) and MedDiet	Cognitive functions, Apolipoprotein E (APOE-4) (risk gene for AD)	HP-EH-EVOO or MP-EVOO was associated with significant improvement in cognitive function compared to MedDiet, independent of the presence of APOE-4	[106]
RCT, prospective, longitudinal, double-blind, MICOIL study on 43 patients with Mild Cognitive Impairment, age: 60–80 years old	HP-EH-EVOO MP-EVOO	1 year of intervention, 3 groups: HP-EH-EVOO (50 mL/day), MP-EVOO (50 mL/day) and MedDiet	Spontaneous Electroencephalography (EEG) dynamic connectivity	Reduced the over excitation of information flow in spontaneous brain activity and increased brain flexibility	[107]
RCT, prospective, longitudinal, double-blind, MICOIL study on 80 patients with Mild Cognitive Impairment, age: 64–80 years old	EVOO	1 year of intervention, 2 groups: with EVOO (50 mL/day) and without EVOO	BMI1, p53, AD biomarkers (tau, p-tau, Aβ1–40, Aβ1–42 and Aβ1–42/Aβ-40 ratio)	The administration of EVOO resulted in an increase of BMI1 and a decrease of p53 and AD biomarkers	[108]
RCT, prospective, longitudinal, double-blind MICOIL study on 84 patients with Mild Cognitive Impairment, age: 65–80 years old	EVOO	1 year of intervention, 2 groups: with EVOO (50 mL/day) and without EVOO	Fibrinolytic system factors (PAI-1, α2AP and tPA), MDA	The administration of EVOO resulted in a reduction of PAI-1, α2AP, tPA, and MDA	[109]
RCT on 25 mild cognitive impaired participants	EVOO ROO	6 months of intervention with a daily intake of 30 mL/day of EVOO (1200 mg/kg total PC; 13 participants) or ROO (no PC; 12 participants)	Clinical dementia rating, behavioral scores, functional connectivity, blood brain barrier connectivity and permeability, brain function, cognitive function, and Alzheimer’s disease blood biomarkers	Daily consumption of EVOO significantly ameliorated the clinical dementia rating and behavioral scores, enhanced the functional connectivity, and decreased the blood–brain barrier permeability. It was concluded that both ROO and EVOO are beneficial with the latter having additional effects due to its bioactive phenolic content.	[110]

BMI: Body Mass Index; DieTBra: Traditional Brazilian Diet; EVOO: Extra-Virgin Olive Oil; HP-EH-EVOO: High-Polyphenol, Early Harvest Extra-Virgin Olive Oil; HQ-MP-EVOO: Moderate-Polyphenol Extra-Virgin Olive Oil; OO: Olive Oil; PC: Phenolic compounds; RCT: Randomized Control Trial; ROO: Refined Olive Oil.

## Data Availability

Not applicable.
